# The novel chimeric multi-agonist peptide (GEP44) reduces energy intake and body weight in male and female diet-induced obese mice in a glucagon-like peptide-1 receptor-dependent manner

**DOI:** 10.3389/fendo.2024.1432928

**Published:** 2024-07-22

**Authors:** James E. Blevins, Mackenzie K. Honeycutt, Jared D. Slattery, Matvey Goldberg, June R. Rambousek, Edison Tsui, Andrew D. Dodson, Kyra A. Shelton, Therese S. Salemeh, Clinton T. Elfers, Kylie S. Chichura, Emily F. Ashlaw, Sakeneh Zraika, Robert P. Doyle, Christian L. Roth

**Affiliations:** ^1^ VA Puget Sound Health Care System, Office of Research and Development Medical Research Service, Department of Veterans Affairs Medical Center, Seattle, WA, United States; ^2^ Division of Metabolism, Endocrinology and Nutrition, Department of Medicine, University of Washington School of Medicine, Seattle, WA, United States; ^3^ Seattle Children’s Research Institute, Seattle, WA, United States; ^4^ Department of Chemistry, Syracuse University, Syracuse, NY, United States; ^5^ Departments of Medicine and Pharmacology, State University of New York (SUNY) Upstate Medical University, Syracuse, NY, United States; ^6^ Department of Pediatrics, University of Washington School of Medicine, Seattle, WA, United States

**Keywords:** obesity, multi-agonist, GLP-1, PYY, IWAT, IBAT

## Abstract

We recently reported that a novel chimeric peptide (GEP44) targeting both the glucagon-like peptide-1 receptor (GLP-1R) and neuropeptide Y1- and Y2 receptor (Y1R and Y2R) reduced energy intake and body weight (BW) in diet-induced obese (DIO) rats. We hypothesized that GEP44 reduces energy intake and BW primarily through a GLP-1R dependent mechanism. To test this hypothesis, GLP-1R^+/+^ mice and GLP-1R null (GLP-1R^-/-^) mice were fed a high fat diet for 4 months to elicit diet-induced obesity prior to undergoing a sequential 3-day vehicle period, 3-day drug treatment (5, 10, 20 or 50 nmol/kg; GEP44 vs the selective GLP-1R agonist, exendin-4) and a 3-day washout. Energy intake, BW, core temperature and activity were measured daily. GEP44 (10, 20 and 50 nmol/kg) reduced BW after 3-day treatment in DIO male GLP-1R^+/+^ mice by -1.5 ± 0.6, -1.3 ± 0.4 and -1.9 ± 0.4 grams, respectively (*P*<0.05), with similar effects being observed in female GLP-1R^+/+^ mice. These effects were absent in male and female DIO GLP-1R^-/-^ mice suggesting that GLP-1R signaling contributes to GEP44-elicited reduction of BW. Further, GEP44 decreased energy intake in both male and female DIO GLP-1R^+/+^ mice, but GEP44 appeared to produce more consistent effects across multiple doses in males. In GLP-1R^-/-^ mice, the effects of GEP44 on energy intake were only observed in males and not females, suggesting that GEP44 may reduce energy intake, in part, through a GLP-1R independent mechanism in males. In addition, GEP44 reduced core temperature and activity in both male and female GLP-1R^+/+^ mice suggesting that it may also reduce energy expenditure. Lastly, we show that GEP44 reduced fasting blood glucose in DIO male and female mice through GLP-1R. Together, these findings support the hypothesis that the chimeric peptide, GEP44, reduces energy intake, BW, core temperature, and glucose levels in male and female DIO mice primarily through a GLP-1R dependent mechanism.

## Introduction

Obesity is a major worldwide health concern as it increases the risk of cardiovascular disease, obstructive sleep apnea, cancer, osteoarthritis, depression, COVID-19 related hospitalizations and type 2 diabetes. According to the NCD Risk Factor Collaboration, more than one billion people are obese worldwide (1). Approximately 1 in 2 US adults are predicted to have obesity by 2030 (2) and the costs to treat obesity in the US are estimated to be approximately 3 trillion/year by 2030 (3). More recently developed monotherapies to treat obesity such as the long-acting glucagon-like peptide-1 receptor (GLP-1R) agonists, liraglutide and semaglutide (4), produce more pronounced effects on weight loss relative to previous analogues. Weight loss in response to once-weekly treatment with semaglutide has ranged from ≈6.7% over 40 weeks (5) to ≈14.9% weight loss over 68 weeks (4). However, improvements still need to be made in terms of improving overall weight loss effectiveness over more prolonged periods [≈10.2% weight loss over 208 weeks (6)]. Furthermore, there is rapid recovery of weight when discontinued (7). This is likely due to the activation of counter-regulatory orexigenic mechanisms that increase energy intake and/or reduce energy expenditure to promote weight regain (8).

Recent studies suggest that combination therapy aimed at suppressing energy intake and/or increasing energy expenditure at low-dose or subthreshold dose combinations may be more effective for producing sustained weight loss than monotherapy (9) and may minimize the potential for unwanted side effects. The overall ineffectiveness of monotherapies to evoke prolonged weight loss in humans with obesity is assumed to occur, in part, by recruitment of robust orexigenic mechanisms that drive energy intake and decrease energy expenditure, resulting in body weight (BW) gain and thus preventing further weight loss. In addition to combination therapy, a recent innovative approach involves the targeting of two or more signaling pathways using a single compound such as monomeric multi-agonists (dual- or triple-agonists) based on glucose-dependent insulinotropic polypeptide (GIP) and GLP-1R agonists, with and without glucagon receptor (GCGR) agonism. One such drug, tirzepatide (Zepbound™), which targets both GLP-1R and and the GIP receptor (GIPR), was found to elicit a robust 20.9% and 25.3% weight loss in humans with obesity over 72- (10) and 88-week periods (11), and was recently approved by the FDA for weight management. Recent data indicate that the triple-agonist, retatrutide, which targets GLP-1R, GIPR and GCGR, was able to elicit 24.2% weight loss over 48-week period (12) (2 to 12 mg). While such therapies show considerable promise for effective and sustained reduction of BW during drug treatment, there are still mild to moderate adverse gastrointestinal side effects including nausea, diarrhea, abdominal pain and vomiting (10). Given the rise of the obesity epidemic in the US and worldwide, there remains an urgent need to develop newer and more effective anti-obesity treatment strategies in order to reduce obesity rates across a broader spectrum of the population. We recently designed the novel chimeric peptide, GEP44, which binds to GLP-1R, Y1R and Y2R (13). We found that systemic administration of GEP44 reduced energy intake and BW in both lean (14) and diet-induced obese rats (13, 14). Importantly, GEP44 also reduced energy intake at doses that were not associated with significant pica behavior (kaolin intake) in rats (14) or emesis in musk shrews (14). A critical remaining question is whether GEP44 reduces energy intake, BW, impacts thermogenesis and improves glucose homeostasis in a GLP-1R dependent manner, either alone or in-part. Given the role of GLP-1R in the control of energy intake and BW, we hypothesized that chimeric peptide GEP44 reduces energy intake and BW through a GLP-1R dependent mechanism. To test this hypothesis, we determined the extent to which GEP44 reduces energy intake and BW, impacts core temperature (core temperature as surrogate marker of energy expenditure) and gross motor activity and improves fasting blood glucose in male and female DIO mice that lack GLP-1R (GLP-1R^-/-^) relative to age-matched cohorts of male and female GLP-1R^+/+^ mice. Male and female mice were also weight-matched within a given cohort of GLP-1R^-/-^ and GLP-1R^+/+^ mice prior to treatment. The selective GLP-1R agonist, exendin-4, was included as a control to assess GLP-1R mediated effects on energy intake (15).

## Methods

### Animals

GLP-1R^+/-^ mice were initially obtained from Dr. Daniel Drucker (University of Toronto, Canada) and bred by Dr. Sakeneh Zraika at the Veterans Affairs Puget Sound Health Care System (VAPSHCS) to obtain GLP-1R^+/+^ and GLP-1R^-/-^ (GLP-1R null) mice. Adult male and female mice (age: ~5.5-10 weeks) weighed, on average, 18.04 ± 0.04 grams and were 10.9 ± 0.03% fat at the time of body composition measurements prior to diet intervention. Mice were initially maintained on a chow diet [PicoLab® Rodent Diet 20 (5053)] (LabDiet®, St. Louis, MO; 13% kcal from fat). Mice were subsequently placed on a high fat diet (HFD) [60% kcal from fat; Research Diets, Inc., D12492i, New Brunswick, NJ] for approximately 4 months at which time body composition measurements were completed. Mice weighed, on average, 39.3 ± 0.04 grams and were 39.8 ± 0.03% fat at the time of body composition measurements prior to drug intervention. All animals were housed individually in Plexiglas cages in a temperature-controlled room (22 ± 2°C) under a 12:12-h light-dark cycle. All mice were maintained on a 11 a.m./11 p.m. reverse light cycle (lights off at 11 a.m./lights on at 11 p.m.). Mice had *ad libitum* access to water and HFD. The research protocols were approved both by the Institutional Animal Care and Use Committee of the Veterans Affairs Puget Sound Health Care System (VAPSHCS) and the University of Washington in accordance with NIH Guidelines for the Care and Use of Animals.

### Drug preparation

GEP44 was synthesized in the Doyle lab as previously described (13). Fresh solutions of GEP44 and exendin-4 (ENZO; Farmingdale, NY) were prepared, frozen and thawed prior to the onset of each experiment (Study 1).

### Implantation of G2 E-Mitter telemetry devices into abdominal cavity

At the age of 27-31.5 weeks (average age 28.8 ± 0.01 weeks), animals were anesthetized with isoflurane and subsequently underwent a sham surgery (no implantation) or received implantations of a sterile G2 E-Mitter (15.5 mm long x 6.5 mm wide; Starr Life Sciences Company) into the intraperitoneal cavity. The abdominal opening was closed with 5-0 Vicryl^®^ absorbable suture and the skin was closed with Nylon sutures (5-0). Vetbond glue was used to seal the wound and bind any tissue together between the sutures. Sutures were removed within two weeks after the G2 E-Mitter implantation. All G2 E-Mitters were confirmed to have remained within the abdominal cavity at the conclusion of the study. Animals were grouped by genotype and sex and matched for body weight (**Table 1**), adiposity and post-surgical weight change prior to drug intervention.

**Table 1:** Body weight (grams) in adult Male and Female GLP-1R^+/+^ and GLP-1R^-/-^ mice post-dietary intervention at study onset.

	GEP44	Exendin-4		GEP44	Exendin-4
**Male WT**	44.1 ± 2.6	43.8 ± 1.9	**Female WT**	32.7 ± 2.8	31.8 ± 1.8
**Male GLP-1R^-/-^**	40.0 ± 1.6	38.0 ± 1.7	**Female GLP1R^-/-^**	28.1 ± 1.2	27.6 ± 1.9

Male GLP-1R^+/+^ GEP44 vs exendin-4 (*P*=NS); Male GLP-1R^-/-^ GEP44 vs exendin-4 (*P*=NS).

Female GLP-1R^+/+^ GEP44 vs exendin-4 (*P*=NS); Female GLP-1R^-/-^ GEP44 vs exendin-4 (*P*=NS).

N=9-11/group.

### Acute SC injections of GEP44 and Exendin-4

GEP44 (or saline vehicle; 3 mL/kg injection volume) or exendin-4 were administered immediately prior to the start of the dark cycle following 2 h of food deprivation. Mice underwent all treatments (unless otherwise noted). The study design consisted of sequential rounds of a 3-day baseline phase (vehicle treated), a 3-day treatment phase (single dose repeated over 3 days), and a washout phase (3 days). The 3-day treatment phase consisted of a dose escalation design beginning with the low dose (5 nmol/kg) over week 1 and ending with the high dose (50 nmol/kg) over the final week of the study (5, 10, 20 and 50 nmol/kg; GEP44 vs the selective GLP-1R agonist, exendin-4). A separate set of age- and weight-matched mice were treated with vehicle in place of drug in order to determine the impact of drug treatment on tail vein glucose, plasma hormones and thermogenic gene expression. BW was assessed daily approximately 3-h prior to the start of the dark cycle.

### Body composition

Determinations of lean body mass and fat mass were made on conscious mice by quantitative magnetic resonance using an EchoMRI 4-in-1-700™ instrument (Echo Medical Systems, Houston, TX) at the VAPSHCS Rodent Metabolic Phenotyping Core.

## Study protocols

### Changes of BW and energy intake

DIO GLP-1R^+/+^ and GLP-1R^-/-^ mice underwent a 3-day vehicle period, 3-day dose escalation drug treatment (5, 10, 20 and 50 nmol/kg; GEP44 vs the selective GLP-1R agonist, exendin-4) and a 3-day washout in sequential order with the same mice receiving the escalating dose. The selective GLP-1R agonist, exendin-4, was included as a control to assess GLP-1R mediated effects on energy intake (15). BW and energy intake were measured daily. BW change reflects the total change over each sequential 3-day vehicle and 3-day drug treatment period. Energy intake data was averaged throughout each sequential 3-day vehicle and 3-day drug treatment period. Note that energy intake was averaged across two-day vehicle treatments (prior to 20 and 50 nmol/kg) for one of the four cohorts used in the dose escalation studies.

### Changes of core temperature and gross motor activity

Telemetry recordings of core temperature (surrogate marker of energy expenditure) and gross motor activity were measured from each mouse in the home cage immediately prior to injections and for a 6-, 12-, and 24-h period after injections. Core temperature and gross motor activity were recorded every 15 sec. The last hour of the light cycle (during which time energy intake, BW and drug administration occurred) was excluded from the telemetry analysis.

Core temperature and activity were averaged throughout each sequential 3-day vehicle and 3-day drug treatment period. Note that core temperature and activity energy intake were averaged across two-day vehicle treatments (prior to 20 and 50 nmol/kg) for one of the four cohorts used in the dose escalation studies.

### Tissue collection for quantitative real-time PCR

Tissue [interscapular brown adipose tissue (IBAT) was collected from 3-h fasted mice at 2-h post-injection. Mice were euthanized with an overdose of ketamine cocktail at 2-h post-injection. Tissue was rapidly removed, wrapped in foil and frozen in liquid N2. Samples were stored frozen at -80°C until analysis. IBAT was collected within a 5-h window towards the end of the light cycle (9:00 a.m.-2:00 p.m.) as previously described in DIO CD® IGS/Long-Evans rats and C57BL/6J mice (16–18).

### Blood collection

Blood samples [up to 1 mL] were collected by cardiac stick in chilled K2 EDTA Microtainer Tubes (Becton-Dickinson, Franklin Lakes, NJ) at 2-h post-injection. Whole blood was centrifuged at 6,000 rpm for 1.5-min at 4°C; plasma was removed, aliquoted and stored at −80°C for subsequent analysis.

### Blood glucose measurements

Blood was collected at 2-h post-injection in a subset of mice for glucose measurements by tail vein nick and measured using a glucometer (AlphaTRAK 2, Abbott Laboratories, Abbott Park, IL) (16, 17).

### Plasma hormone measurements

Plasma leptin, insulin and glucagon were measured using electrochemiluminescence detection [leptin/insulin: Meso Scale Discovery (MSD^®^), Rockville, MD] or (glucagon: Mercodia, Uppsala, Sweden) using established procedures (17, 19). Intra-assay coefficient of variation (CV) were 5.9%, 1.6% and 5.7% for leptin, insulin and glucagon. The range of detectability for the assays are as follows: 0.137-100 ng/mL (leptin), 0.069-50 ng/mL (insulin) and 0.002-0.183 ng/mL (glucagon). The data were normalized to historical values using a pooled plasma quality control sample that was assayed in each plate.

### qPCR

RNA extracted from samples of IBAT was analyzed using the RNeasy Lipid Mini Kit (Qiagen Sciences Inc, Germantown, MD) followed by reverse transcription into cDNA using a high-capacity cDNA archive kit (Applied Biosystems, Foster City, CA). Quantitative analysis for relative levels of mRNA in the RNA extracts was measured in duplicate by qPCR on an Applied Biosystems 7500 Real-Time PCR system (Thermo Fisher Scientific, Waltham, MA) using the following TaqMan^®^ probes (Thermo Fisher Scientific Gene Expression Assay probes): mouse Nono (catalog no. Mm00834875_g1), mouse UCP-1 (catalog no. Mm01244861_m1), mouse type 2 deiodinase (D2) (Dio2; catalog no. Mm00515664_m1), mouse G-protein coupled receptor 120 (Gpr120; catalog no. Mm00725193_m1), mouse cell death-inducing DNA fragmentation factor alpha-like effector A (Cidea; catalog no. Mm00432554_m1) and mouse peroxisome proliferator-activated receptor gamma coactivator 1 alpha (Ppargc1a; catalog no. Mm01208835_m1). Relative amounts of target mRNA were determined using the Comparative C_T_ or 2-^ΔΔC^_T_ method (20) following adjustment for the housekeeping gene, Nono. Specific mRNA levels of all genes of interest were normalized to the cycle threshold value of Nono mRNA in each sample and expressed as changes normalized to controls (vehicle treatment).

## Statistical analyses

All results are expressed as mean ± SE. Planned comparisons within respective genotypes and sex between vehicle and drug (plasma measures, tail vein glucose, and gene expression data) involving between subjects design were made using one-way ANOVA. Planned comparisons within respective genotypes and sex to examine treatment means of vehicle and drug (BW change, energy intake, core temperature, activity and T_IBAT_) involving within-subjects designs were made using a one-way repeated-measures ANOVA. A two-way ANOVA was used to examine sex*drug interactive effects on BW change and energy intake. In addition, planned comparisons were used to examine sex differences on BW change and energy intake using a one-way ANOVA. Analyses were performed using the statistical program SYSTAT (Systat Software, Point Richmond, CA). Differences were considered significant at *P*<0.05, 2-tailed.

## Results

The overall goal of these studies was to use the GLP-1R^-/-^ mouse as a strategy to determine the extent to which chimeric peptide, GEP44, reduces BW and energy intake, impacts core temperature (as surrogate for energy expenditure) and activity, and improves glucose homeostasis through the GLP-1R in both male and female DIO mice. In addition, we incorporated use of the selective GLP-1R agonist, exendin-4, to assess GLP-1R mediated effects on BW, energy intake, core temperature, activity, and glucose levels in male and female DIO mice. DIO GLP-1R^+/+^ and GLP-1R^-/-^ mice underwent a 3-day vehicle period, 3-day dose escalation drug treatment (5, 10, 20 and 50 nmol/kg; GEP44 vs the selective GLP-1R agonist, exendin-4) and a 3-day washout in sequential order with the same mice receiving the escalating dose. Energy intake, core temperature and activity were averaged throughout each sequential 3-day vehicle and 3-day drug treatment period. Note that energy intake, core temperature and activity were averaged across two-day vehicle treatments (prior to 20 and 50 nmol/kg) for one of the four cohorts used in the dose escalation studies.

Baseline BW-matching in Male and Female DIO GLP-1R^+/+^ and GLP-1R^-/-^ Mice at Study Onset (post-dietary intervention/pre-drug treatment). By design, there were no differences in baseline BW between designated drug treatment groups (GEP44 vs exendin-4) of male GLP-1R^+/+^ mice [F(1,19) = 0.009, *P*=NS] or male GLP-1R^-/-^ mice [F(1,18) = 0.682, *P*=NS] prior to drug treatment (**Table 1**). Similarly, there were also no differences in baseline BW between designated treatment groups (GEP44 vs exendin-4) of female GLP-1R^+/+^ mice [F(1,18) = 0.071, *P*=NS] or GLP-1R^-/-^ mice [F(1,16) = 0.037, P=NS] prior to drug treatment (**Table 1**).

### Body weight

**Figure 1:**
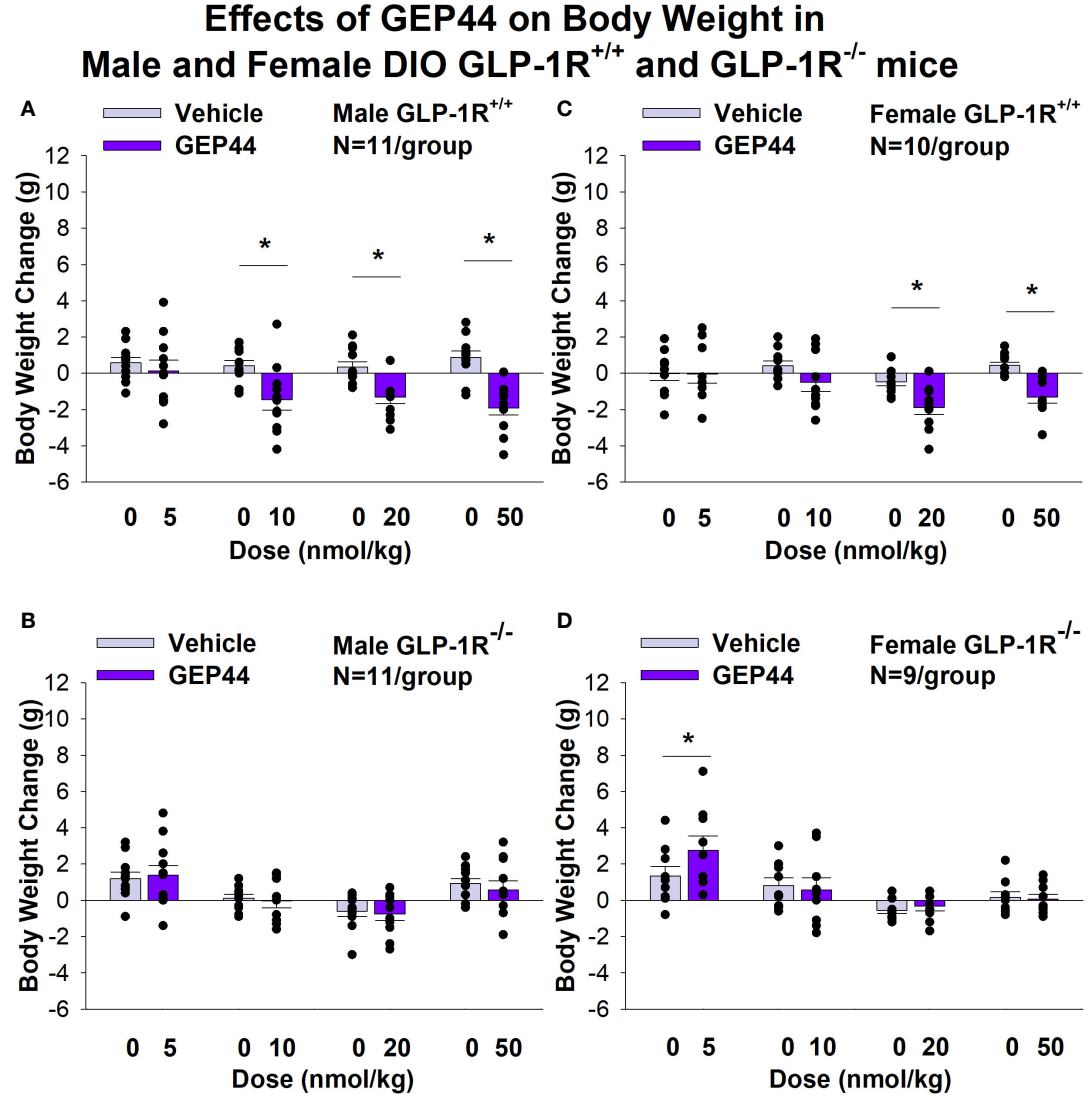
**(A–D)** Effects of the chimeric peptide, GEP44, on BW in male and female DIO GLP-1R^+/+^ and GLP-1R^-/-^ mice. Mice were maintained on HFD (60% kcal from fat; N=10-11/group) for approximately 4 months prior to receiving SC injections of vehicle (sterile saline/water) followed by escalating doses of GEP44 (5, 10, 20 and 50 nmol/kg; 3 mL/kg injection volume). **(A)** Effect of GEP44 on change in BW in male HFD-fed DIO GLP-1R^+/+^ mice; **(B)** Effect of GEP44 on change in BW in male HFD-fed DIO GLP-1R ^-/-^ mice; **(C)** Effect of GEP44 on change in BW in female HFD-fed DIO GLP-1R^+/+^ mice; **(D)** Effect of GEP44 on change in BW in female HFD-fed DIO GLP-1R^-/-^ mice. BW change reflects the total change over each sequential 3-day vehicle and 3-day drug treatment period. Data are expressed as mean ± SEM. *P<0.05 GEP44 vs. vehicle.

Both male and female GLP-1R^+/+^ and GLP-1R^-/-^ mice were weight-matched within respective groups prior to treatment onset (**Table 1**). GEP44 treatment reduced cumulative 3-day BW in both male (**Figures 1A, B**) and female DIO GLP-1R^+/+^ mice (**Figures 1C, D**), but these effects were absent in male and female DIO GLP-1R^-/-^ mice. Specifically, GEP44 (10, 20 and 50 nmol/kg), reduced 3-day BW in DIO male GLP-1R^+/+^ mice by -1.5 ± 0.6, -1.3 ± 0.4 and -1.9 ± 0.4 grams, respectively (**Figures 1A, B**; *P*<0.05). GEP44 also reduced 3-day BW (10, 20 and 50 nmol/kg) in female GLP-1R^+/+^ mice by -0.5 ± 0.5 (*P*=0.053), -1.9 ± 0.4 and -1.3 ± 0.3 grams, respectively (**Figures 1C, D**; *P*<0.05).

**Figure 2:**
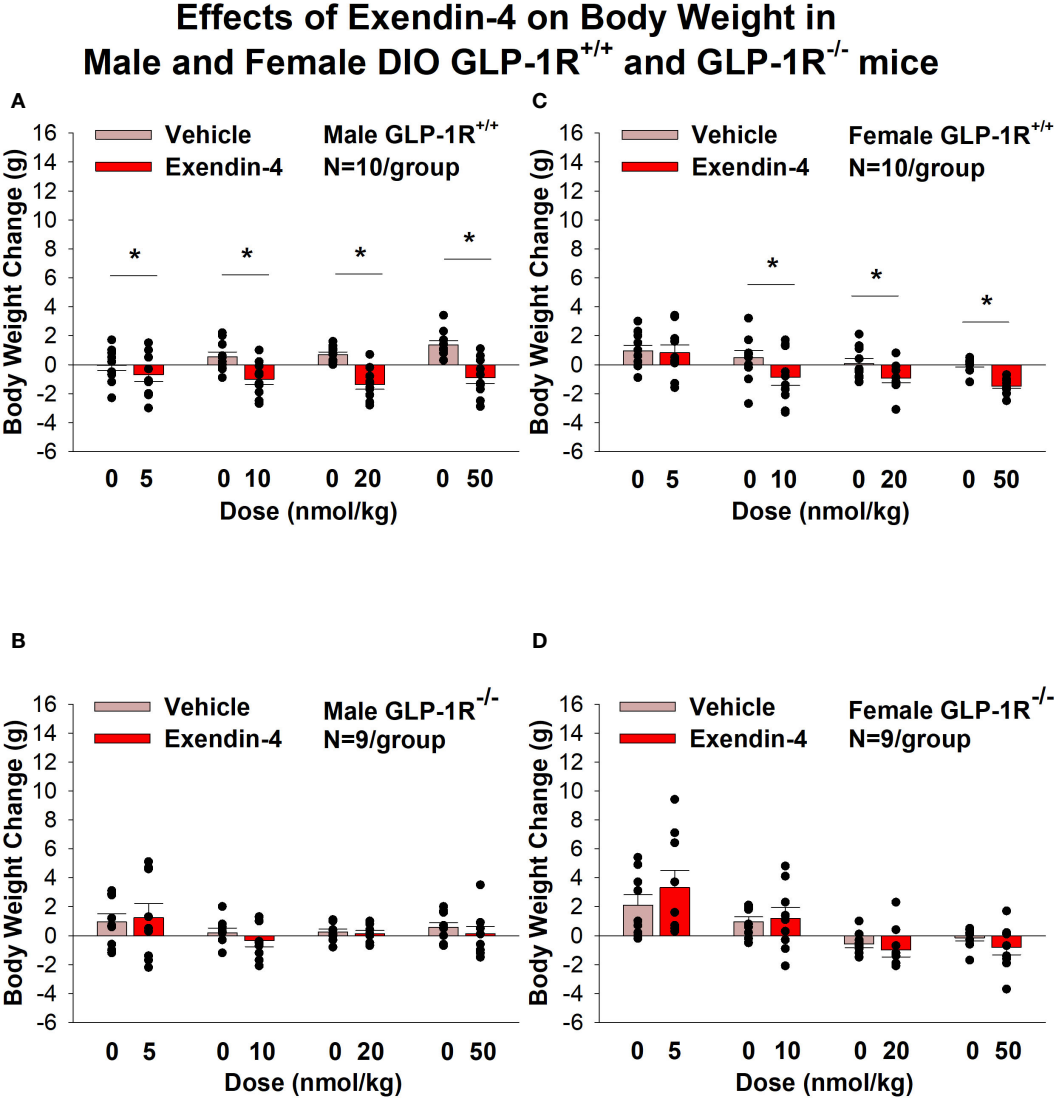
**(A–D)** Effects of the selective GLP-1R agonist, exendin-4, on BW in male and female DIO GLP-1R^+/+^ and GLP-1R^-/-^ mice. Mice were maintained on HFD (60% kcal from fat; N=9-11/group) for approximately 4 months prior to receiving SC injections of vehicle (sterile saline/water) followed by escalating doses of exendin-4 (5, 10, 20 and 50 nmol/kg; 3 mL/kg injection volume). **(A)** Effect of exendin-4 on change in BW in male HFD-fed DIO GLP-1R^+/+^ mice; **(B)** Effect of exendin-4 on change in BW in male HFD-fed DIO GLP-1R^-/-^ mice; **(C)** Effect of exendin-4 on change in BW in female HFD-fed DIO GLP-1R^+/+^ mice; **(D)** Effect of exendin-4 on change in BW in female HFD-fed DIO GLP-1R^-/-^ mice. BW change reflects the total change over each sequential 3-day vehicle and 3-day drug treatment period. Data are expressed as mean ± SEM. *P<0.05 exendin-4 vs. vehicle.

Similarly, the selective GLP-1R agonist, exendin-4, reduced BW only in male (**Figures 2A, B**; *P*<0.05) and female DIO GLP-1R^+/+^ mice (**Figures 2C, D**; *P*<.05) but not in male and female DIO GLP-1R ^-/-^ mice. Specifically, exendin-4 (5, 10, 20 and 50 nmol/kg) reduced BW in DIO male GLP-1R^+/+^ mice by -0.7 ± 0.5, -1.0 ± 0.4, -1.4 ± 0.3, and -1.5 ± 0.2 grams, respectively (**Figures 1A, B**; *P*<0.05). Exendin-4 also reduced BW (10, 20 and 50 nmol/kg) in female GLP-1R^+/+^ mice by -0.9 ± 0.6, -0.9 ± 0.3 and -1.5 ± 0.2 grams, respectively (**Figures 1C, D**; *P*<0.05).

## Energy intake

**Figure 3:**
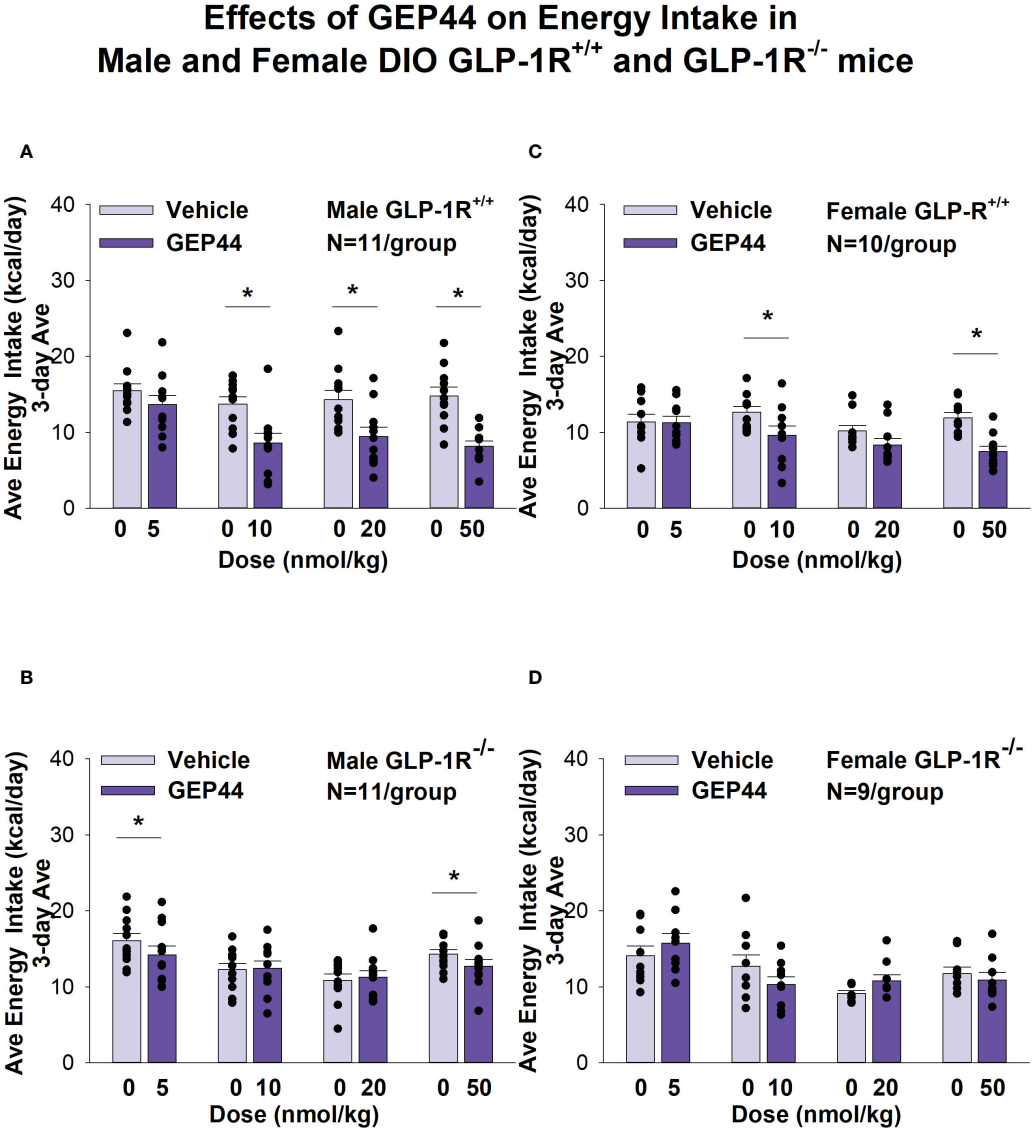
**(A–D)** Effects of the chimeric peptide, GEP44, on energy intake (kcal/day) in male and female DIO GLP-1R^+/+^ and GLP-1R ^-/-^ mice. Mice were maintained on HFD (60% kcal from fat; N=10-11/group) for approximately 4 months prior to receiving SC injections of vehicle (sterile saline/water) followed by escalating doses of GEP44 (5, 10, 20 and 50 nmol/kg; 3 mL/kg injection volume). **(A)** Effect of GEP44 on energy intake in male HFD-fed DIO GLP-1R^+/+^ mice; **(B)** Effect of GEP44 on change on energy intake in male HFD-fed DIO GLP-1R^-/-^ mice; **(C)** Effect of GEP44 on energy intake in male HFD-fed DIO GLP-1R^+/+^ mice; **(D)** Effect of GEP44 on energy intake in male HFD-fed DIO GLP-1R^-/-^ mice. Energy intake was averaged throughout each sequential 3-day vehicle and 3-day drug treatment period. Data are expressed as mean ± SEM. *P<0.05 GEP44 vs. vehicle.

GEP44 treatment significantly reduced energy intake only in male (**Figures 3A, B**) and female DIO GLP-1R^+/+^ mice (**Figures 3C, D**) but not in male (with exception of the lowest and highest dose) and female DIO GLP-1R ^-/-^ mice. GEP44 treatment decreased energy intake (10, 20 and 50 nmol/kg); *P*<0.005) in both male and female mice. Similar results on FI were obtained when normalizing to BW (**Supplementary Figures 1A–D**).

**Figure 4:**
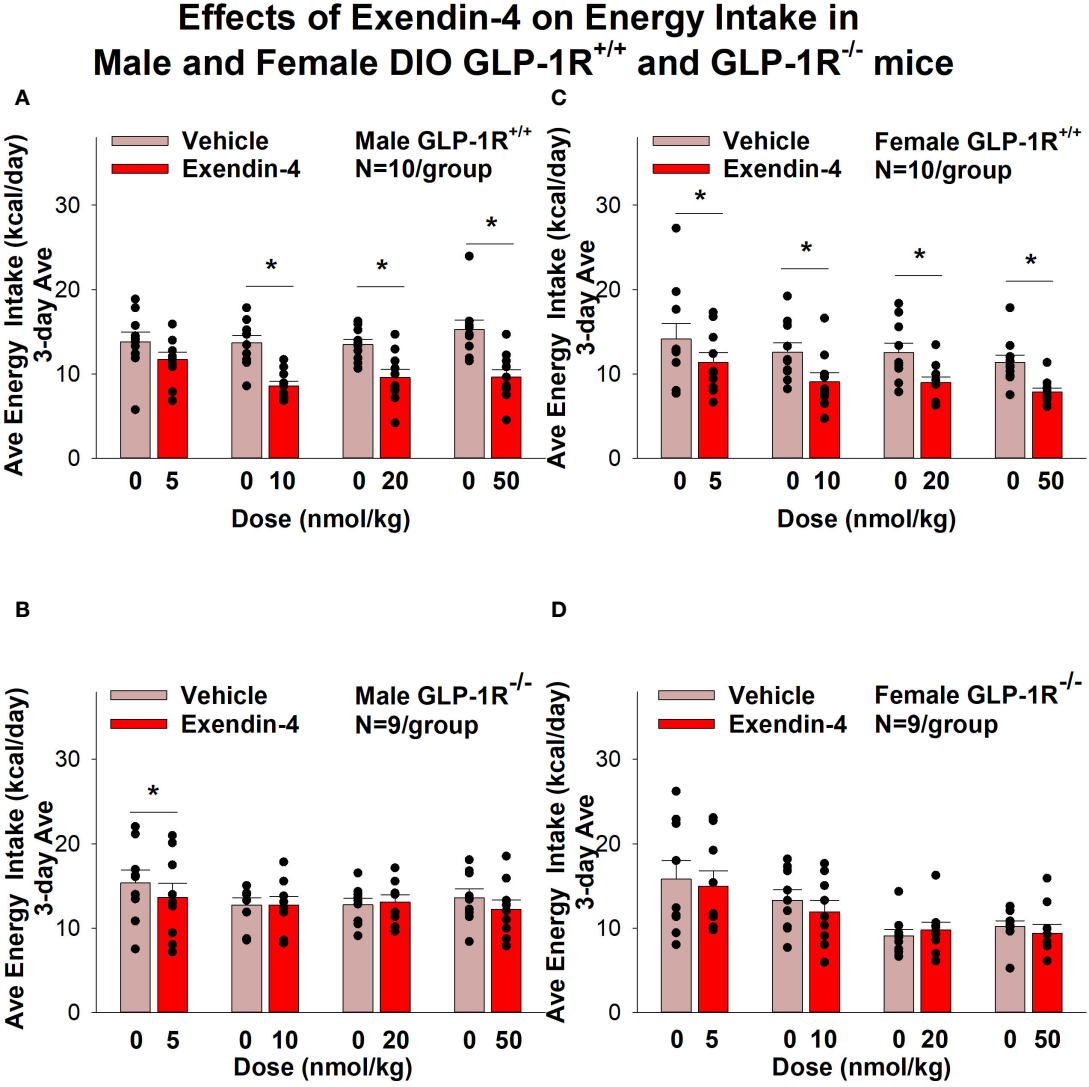
**(A–D)** Effects of the selective GLP-1R agonist, exendin-4, on energy intake (kcal/day) in male and female DIO GLP-1R^+/+^ and GLP-1^-/-^ mice. Mice were maintained on HFD (60% kcal from fat; N=9-11/group) for approximately 4 months prior to receiving SC injections of vehicle followed by escalating doses of GEP44 (5, 10, 20 and 50 nmol/kg; 3 mL/kg injection volume). **(A)** Effect of GEP44 on energy intake in male HFD-fed DIO GLP-1R^+/+^ mice; **(B)** Effect of GEP44 on change on energy intake in male HFD-fed DIO GLP-1R^-/-^ mice; **(C)** Effect of GEP44 on energy intake in male HFD-fed DIO GLP-1R^+/+^ mice; **(D)** Effect of GEP44 on energy intake in male HFD-fed DIO GLP-1R^-/-^ mice. Energy intake was averaged throughout each sequential 3-day vehicle and 3-day drug treatment period. Data are expressed as mean ± SEM. *P<0.05 exendin-4 vs. vehicle.

Exendin-4 treatment also significantly reduced energy intake in male (**Figures 4A, B**) and female DIO GLP-1R^+/+^ mice (**Figures 4C, D**) but not in male (with exception of the lowest dose) and female DIO GLP-1R ^-/-^ mice. Specifically, exendin-4 treatment decreased energy intake (10, 20 and 50 nmol/kg; *P*<0.05) in male GLP-1R^+/+^ mice and also tended to decrease energy intake at the low dose (5 nmol/kg) in male GLP-1R ^-/-^ mice (*P*=0.055). However, exendin-4 reduced energy intake in female GLP-1R^+/+^ mice across all doses (5, 10, 20 and 50 nmol/kg; *P*<0.05).

Similar results on energy intake were obtained when normalizing to BW (**Supplementary Figures 2A–D**).

### Sex differences linked to effects of GEP44 and Exendin-4 on body weight and energy intake

There was a significant difference in the effectiveness of GEP44 to suppress energy intake in male and female GLP-1R^+/+^ mice with males showing the more pronounced effect of GEP44 to reduce energy intake at both 20 [F(1,19)= 6.524, *P*=0.019] and 50 nmol/kg [F(1,13)= 4.880, *P*=0.046]. Two-way repeated-measures ANOVA revealed a significant effect of sex [F(1,26) = 8.292, *P*=0.008] and treatment with GEP44 (20 nmol/kg) [F(1,26)= 32.110, *P*<0.01] on energy intake and a near significant interaction between sex and GEP44 on energy intake [F(1,26) = 3.112, *P*=0.089]. At the higher dose (50 nmol/kg) of GEP44, there was also a near significant effect of sex [F(1,26) = 3.902, *P*=0.059] and treatment with GEP44 [F(1,26) = 48.490, *P*<0.01] on energy intake but there was no significant interactive effect between sex and GEP44.

There was also a significant difference in the effectiveness of exendin-4 (5 nmol/kg) to reduce BW in male and female GLP-1R^+/+^ mice with males showing the more heightened effect [F(1,18) = 4.698, *P*=0.044]. Two-way ANOVA revealed, however, that despite the overall effect of sex [(F1,26) = 6.954, *P*=0.014], there was no overall significant effect of exendin-4 treatment [(F1,26) = 1.052, *P*=0.314] and no interactive effect between sex and exendin-4 treatment [(F1,26) = 0.460, *P*=0.504].

### Changes of core temperature and gross motor activity

The analysis focused on the effects of GEP44 and exendin-4 on core temperature and activity at 6-h post-injection (dark cycle). We also discuss the extent to which these effects carried into the 12-h dark cycle, 11-h light cycle and 23-h post-injection period. Note that the last hour of the light cycle (hour 12) was excluded from the core temperature and activity analysis as this was during the time that the animals were being handled and injected.

#### Core temperature

**Figure 5:**
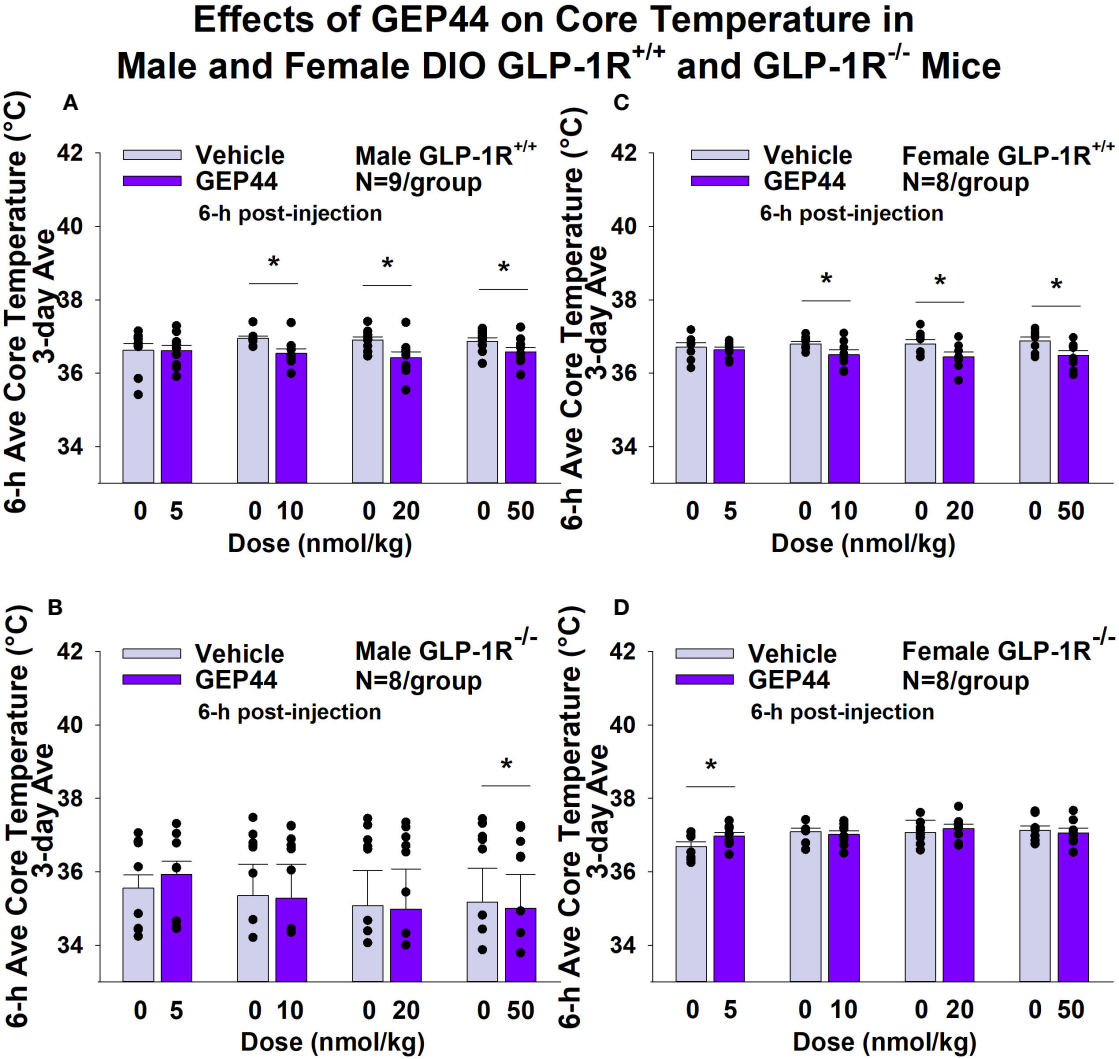
**(A–D)**. Effects of the chimeric peptide, GEP44, on core temperature in male and female DIO GLP-1R^+/+^ and GLP-1R^-/-^ mice. Mice were maintained on HFD (60% kcal from fat; N=8-9/group) for approximately 4 months prior to receiving SC injections of vehicle (sterile saline/water) followed by escalating doses of GEP44 (5, 10, 20 and 50 nmol/kg; 3 mL/kg injection volume). **(A)** Effect of GEP44 on change in core temperature in male HFD-fed DIO GLP-1R^+/+^ mice; **(B)** Effect of GEP44 on change in core temperature in male HFD-fed DIO GLP-1R ^-/-^ mice; **(C)** Effect of GEP44 on change in core temperature in female HFD-fed DIO GLP-1R^+/+^ mice; **(D)** Effect of GEP44 on change in core temperature in female HFD-fed DIO GLP-1R^-/-^ mice. Core temperature was averaged throughout each sequential 3-day vehicle and 3-day drug treatment period. Data are expressed as mean ± SEM. *P<0.05 GEP44 vs. vehicle.

**Figure 6:**
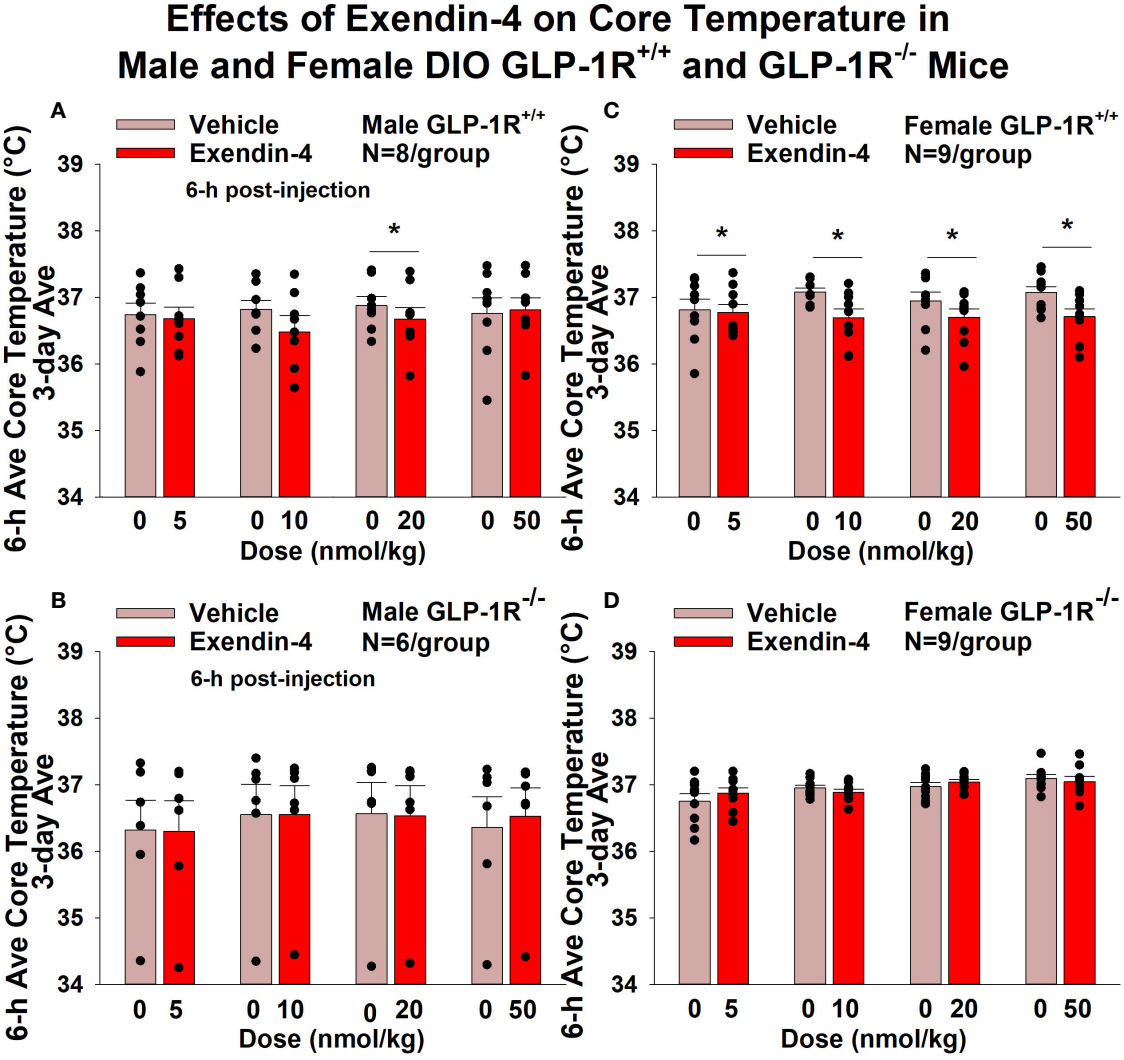
**(A–D)** Effects of the selective GLP-1R agonist, exendin-4, on core temperature in male and female DIO GLP-1R^+/+^ and GLP-1R^-/-^ mice. Mice were maintained on HFD (60% kcal from fat; N=6-9/group) for approximately 4 months prior to receiving SC injections of vehicle (sterile saline/water) followed by escalating doses of GEP44 (5, 10, 20 and 50 nmol/kg; 3 mL/kg injection volume). **(A)** Effect of exendin-4 on change in core temperature in male HFD-fed DIO GLP-1R^+/+^ mice; **(B)** Effect of exendin-4 on change in core temperature in male HFD-fed DIO GLP-1R^-/-^ mice; **(C)** Effect of exendin-4 on change in core temperature in female HFD-fed DIO GLP-1R^+/+^ mice; **(D)** Effect of exendin-4 on change in core temperature in female HFD-fed DIO GLP-1R^-/-^ mice. Core temperature was averaged throughout each sequential 3-day vehicle and 3-day drug treatment period. Data are expressed as mean ± SEM. *P<0.05 exendin-4 vs. vehicle.

GEP44 and exendin-4 both reduced core temperature over the 6-h post-injection period in male (**Figure 5A**; *P*<0.05) and female DIO GLP-1R^+/+^ mice (**Figure 5C**; *P*<0.05). Notably, similar results were observed during the 12-h dark cycle in response to GEP44 (10 and 20 nmol/kg) in male DIO GLP-1R^+/+^ mice (*P*<0.05; data not shown). GEP44 (10 nmol/kg) also reduced core temperature in male GLP-1R^+/+^ mice during the 23-h post-injection period (*P*<0.05; data not shown). However, the effect of GEP44 to reduce core temperature was observed only at the high dose in male GLP-1R^-/-^ mice (50 nmol/kg; **Figure 5B**; *P*<0.05). GEP44 was unable to reduce core temperature in female GLP-1R^-/-^ mice (**Figure 5D**; *P*=NS). However, GEP44 produced an unexpected stimulation of core temperature at the low dose in female GLP-1R^-/-^ mice (**Figure 5D**; *P*<0.05). There was an effect of exendin-4 to reduce core temperature in male (**Figure 6A**; *P*<0.05) or female GLP-1R^+/+^ mice (**Figure 6C**; *P*<0.05) but not in GLP-1R^-/-^ mice (**Figures 6B**, **D** *; P*=NS). Exendin-4 was largely without effect on core temperature during the 12-h dark, 11-h light and 23-h post-injection period in male DIO GLP-1R^+/+^ mice. It did, however, produce a slight elevation of core temperature during the light cycle (20 nmol/kg; *P*<0.05). GEP44 produced opposing effects on core temperature during the dark (decrease) and light (increase) cycles (20 nmol/kg) but was otherwise ineffective at altering core temperature during the 12-h dark, 11-h light and 23-h post-injection period in female DIO GLP-1R^+/+^ mice (*P*=NS).

#### Activity

**Figure 7:**
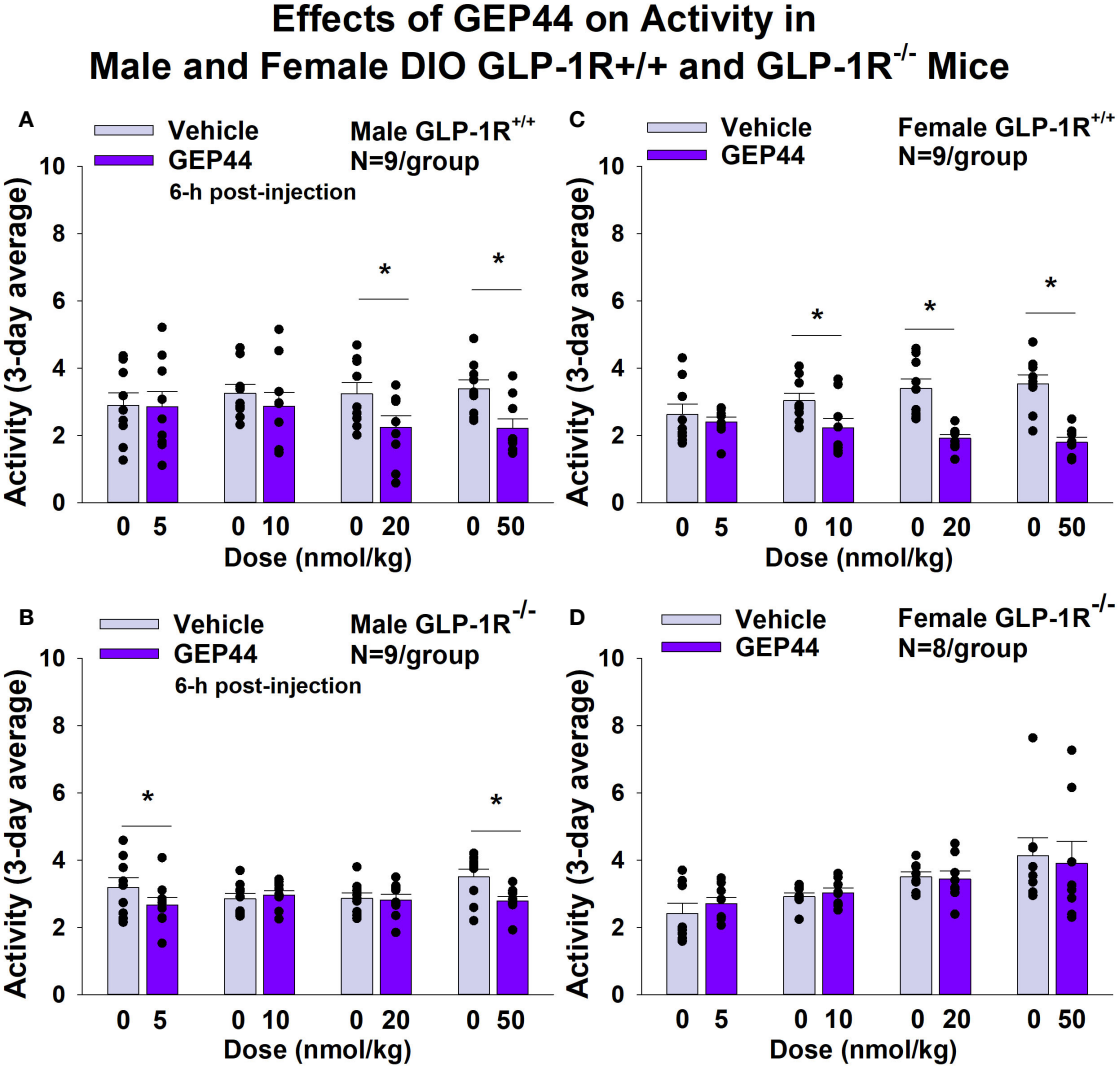
**(A–D)**. Effects of the chimeric peptide, GEP44, on activity in male and female DIO GLP-1R^+/+^ and GLP-1R^-/-^ mice. Mice were maintained on HFD (60% kcal from fat; N=7-9/group) for approximately 4 months prior to receiving SC injections of vehicle (sterile saline/water) followed by escalating doses of GEP44 (5, 10, 20 and 50 nmol/kg; 3 mL/kg injection volume). **(A)** Effect of GEP44 on change in activity in male HFD-fed DIO GLP-1R^+/+^ mice; **(B)** Effect of GEP44 on change in activity in male HFD-fed DIO GLP-1R^-/-^ mice; **(C)** Effect of GEP44 on change in activity in female HFD-fed DIO GLP-1R^+/+^ mice; **(D)** Effect of GEP44 on change in activity in female HFD-fed DIO GLP-1R^-/-^ mice. Activity was averaged throughout each sequential 3-day vehicle and 3-day drug treatment period. Data are expressed as mean ± SEM. *P<0.05 GEP44 vs. vehicle.

**Figure 8:**
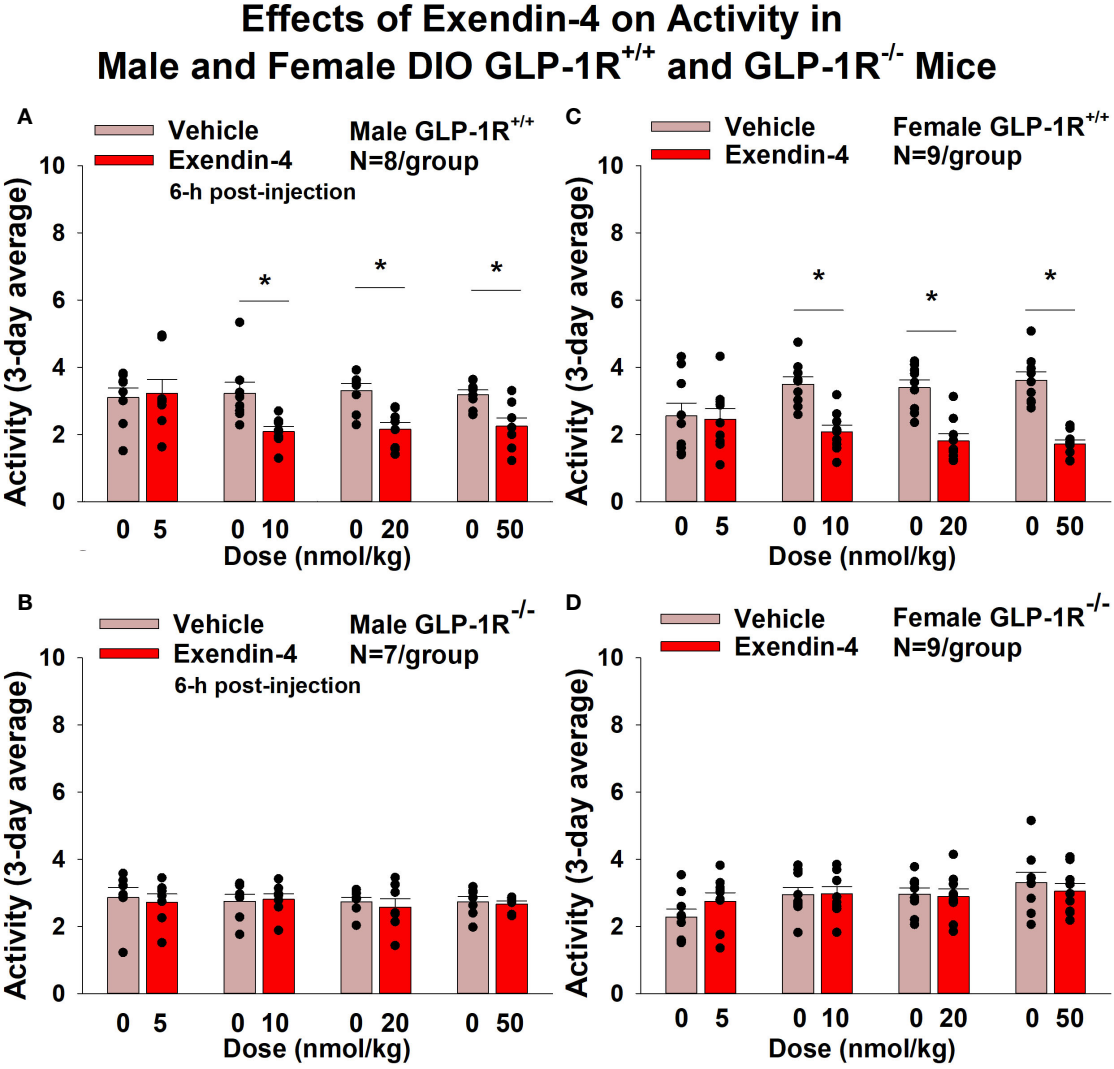
**(A–D)** Effects of the selective GLP-1R agonist, exendin-4, on activity in male and female DIO GLP-1R^+/+^ and GLP-1R^-/-^ mice. Mice were maintained on HFD (60% kcal from fat; N=8-9/group) for approximately 4 months prior to receiving SC injections of vehicle (sterile saline/water) followed by escalating doses of GEP44 (5, 10, 20 and 50 nmol/kg; 3 mL/kg injection volume). **(A)** Effect of exendin-4 on change in activity in male HFD-fed DIO GLP-1R^+/+^ mice; **(B)** Effect of exendin-4 on change in activity in male HFD-fed DIO GLP-1R^-/-^ mice; **(C)** Effect of exendin-4 on change in activity in female HFD-fed DIO GLP-1R^+/+^ mice; **(D)** Effect of exendin-4 on change in activity in female HFD-fed DIO GLP-1R^-/-^ mice. Activity was averaged throughout each sequential 3-day vehicle and 3-day drug treatment period. Data are expressed as mean ± SEM. *P<0.05 exendin-4 vs. vehicle.

GEP44 and exendin-4 both reduced activity over 6-h post-injection in male (**Figure 7A**; *P*<0.05) and female DIO GLP-1R^+/+^ mice (**Figure 7C**; *P*<0.05). Similar results also observed over the 12-h dark cycle in response to GEP44 in male and female DIO GLP-1R^+/+^ mice (*P*<0.05; data not shown). GEP44 (50 nmol/kg) also reduced activity during the 12-h dark and 23-h post-injection period in female DIO GLP-1R^+/+^ mice (*P*<0.05) but had no effect during the 11-h light post-injection period. The lower dose of GEP44 (20 nmol/kg) reduced activity only during the 12-h dark period (*P*<0.05). In addition, GEP44 continued to reduce core temperature during the 12-h dark (10 nmol/kg, 20 and 50 nmol/kg), 11-h light (5 nmol/kg) and 23-h post-injection periods (5, 10, and 50 nmol/kg) in female DIO GLP-1R^+/+^ mice (*P*<0.05). However, the effect of GEP44 to reduce activity was observed only at the low and high dose in male GLP-1R^-/-^ mice (**Figure 7B**; *P*<0.05) and not in female GLP-1R^-/-^ mice (**Figure 7D**; *P*=NS). There was an effect of exendin-4 to reduce activity in male (**Figure 8A**; *P*<0.05) and female GLP-1R^+/+^ mice (**Figure 8C**; *P*=NS) but not in GLP-1R^-/-^ mice (**Figures 8B**, **D**; *P*=NS). Exendin-4 had no effect on activity in male GLP-1R^+/+^ during the 12-h dark, 11-h light or 23-h post-injection period. In contrast, exendin-4 reduced activity in female GLP-1R^+/+^ during the 12-h dark (20 and 50 nmol/kg; *P*<0.05), 11-h light [5 nmol/kg (*P*><0.05) and 20 nmol/kg (*P*=0.050)], and 23-h post-injection period (20 and 50 nmol/kg; *P*<0.05).

### Tissue collection for quantitative real-time PCR

As an additional readout of GEP44 and exendin-4-elicited thermogenic effects in IBAT, relative levels of mRNA for UCP-1, Gpr120, Ppargc1a, Cidea, and Dio2 were compared by PCR in response to GEP44 (50 nmol/kg) and exendin-4 (50 nmol/kg) or vehicle treatment at 2-h post-injection in male (**Table 2A**) and female GLP-1R^+/+^ mice (**Table 2C**). We found that GEP44 increased Ppargc1a in male GLP-1R^+/+^ mice [F(1,20) = 7.506, *P*=0.013]. Similarly, GEP44 increased Ppargc1a in female GLP-1R^+/+^ mice [F(1,18) = 36.773, P<0.001]. In contrast, the effects of GEP44 on thermogenic gene expression were largely absent in male GLP-1R^-/-^ mice (**Table 2B**). In addition, GEP44 stimulated GPR120 in female GLP-1R^-/-^ mice (**Table 2D**).

Table 2(A–D) Changes in IBAT gene expression following GEP44 and Exendin-4 treatment in male and female DIO GLP-1R^+/+^ and GLP-1R^-/-^ mice.

**Table 2A:** Changes in IBAT gene expression following GEP44 and Exendin-4 in male DIO GLP-1R^+/+^ mice.

DIO GLP-1R^+/+^ Mice				
Treatment	VEH	GEP44	VEH	Exendin-4
IBAT				
*UCP1*	1.0 ± 0.2^a^	1.9 ± 0.5^a^	1.0 ± 0.1^a^	1.6 ± 0.4^a^
*Gpr120*	1.0 ± 0.2^a^	1.8 ± 0.4^a^	1.0 ± 0.2^a^	1.2 ± 0.3^a^
*Ppargc1a*	1.0 ± 0.1^a^	1.7 ± 0.2^b^	1.0 ± 0.1^a^	1.3 ± 0.2^a^
*Cidea*	1.0 ± 0.2^a^	1.2 ± 0.2^a^	1.0 ± 0.2^a^	1.6 ± 0.6^a^
*Dio2*	1.0 ± 0.3^a^	0.8 ± 0.3^a^	1.0 ± 0.3^a^	0.7 ± 0.2^a^

Different letters denote significant differences between treatments (VEH vs GEP44/VEH vs Exendin-4; *P*<0.05).Shared letters are not significantly different from one another (VEH vs GEP44/VEH vs Exendin-4).IBAT was collected at 2-h post-injection of VEH, exendin-4 (50 nmol/kg) or GEP44 (50 nmol/kg).Data are expressed as mean ± SEM.N=5-12/group.

**Table 2B:** Changes in IBAT gene expression following GEP44 and Exendin-4 in male DIO GLP-1R^-/-^ mice.

DIO GLP-1R^-/-^ Mice				
Treatment	VEH	GEP44	VEH	Exendin-4
IBAT				
*UCP1*	1.0 ± 0.2^a^	1.7± 0.4^a^	1.0 ± 0.2^a^	1.7± 0.5^a^
*Gpr120*	1.0 ± 0.3^a^	1.0 ± 0.2^a^	1.0 ± 0.3^a^	1.2 ± 0.3^a^
*Ppargc1a*	1.0 ± 0.2^a^	1.2 ± 0.1^a^	1.0 ± 0.2^a^	1.6 ± 0.4^a^
*Cidea*	1.0 ± 0.3^a^	1.4 ± 0.3^a^	1.0 ± 0.3^a^	1.8 ± 0.4^a^
*Dio2*	1.0 ± 0.4^a^	0.9 ± 0.2^a^	1.0 ± 0.4^a^	1.2 ± 0.4^a^

Different letters denote significant differences between treatments (VEH vs GEP44/VEH vs Exendin-4; *P*<0.05).Shared letters are not significantly different from one another (VEH vs GEP44/VEH vs Exendin-4).IBAT was collected at 2-h post-injection of VEH, exendin-4 (50 nmol/kg) or GEP44 (50 nmol/kg).Data are expressed as mean ± SEM. N=4-11/group.

**Table 2C:** Changes in IBAT gene expression following GEP44 and Exendin-4 in female DIO GLP-1R^+/+^ mice.

DIO GLP-1R^+/+^ Mice				
Treatment	VEH	GEP44	VEH	Exendin-4
IBAT				
*UCP1*	1.0 ± 0.1^a^	1.4 ± 0.2^a^	1.0 ± 0.1^a^	1.9 ± 0.4^b^
*Gpr120*	1.0 ± 0.2^a^	1.7 ± 0.4^a^	1.0 ± 0.2^a^	2.7 ± 0.5^b^
*Ppargc1a*	1.0 ± 0.1^a^	2.1 ± 0.2^b^	1.0 ± 0.1^a^	2.7 ± 0.4^b^
*Cidea*	1.0 ± 0.2^a^	0.8 ± 0.2^a^	1.0 ± 0.2^a^	1.6 ± 0.6^a^
*Dio2*	1.0 ± 0.2^a^	1.4 ± 0.4^a^	1.0 ± 0.3^a^	0.7 ± 0.2^a^

Different letters denote significant differences between treatments (VEH vs GEP44 or Exendin-4; *P*<0.05).Shared letters are not significantly different from one another (VEH vs GEP44 or Exendin-4).IBAT was collected at 2-h post-injection of VEH, exendin-4 (50 nmol/kg) or GEP44 (50 nmol/kg).Data are expressed as mean ± SEM. *P<0.05 GEP44 or exendin-4 vs. vehicle.N=3-10/group.

**Table 2D:** Changes in IBAT gene expression following GEP44 and Exendin-4 in female DIO GLP-1R^-/-^ mice.

DIO GLP-1R^-/-^ Mice				
Treatment	VEH	GEP44	VEH	Exendin-4
IBAT				
*UCP1*	1.0 ± 0.1^a^	1.9 ± 0.5^a^	1.0 ± 0.1^a^	1.7 ± 0.3^a^
*Gpr120*	1.0 ± 0.2^a^	2.0 ± 0.4^b^	1.0 ± 0.2^a^	1.5 ± 0.3^a^
*Ppargc1a*	1.0 ± 0.1^a^	1.1 ± 0.1^a^	1.0 ± 0.1^a^	1.4 ± 0.1^a^
*Cidea*	1.0 ± 0.2^a^	1.3 ± 0.2^a^	1.0 ± 0.2^a^	2.0 ± 0.6^a^
*Dio2*	1.0 ± 0.2^a^	1.6 ± 0.7^a^	1.0 ± 0.2^a^	1.5 ± 0.3^a^

Different letters denote significant differences between treatments (VEH vs GEP44/VEH vs Exendin-4; *P*<0.05).Shared letters are not significantly different from one another (VEH vs GEP44/VEH vs Exendin-4).IBAT was collected at 2-h post-injection of VEH, exendin-4 (50 nmol/kg) or GEP44 (50 nmol/kg).Data are expressed as mean ± SEM. N=3-9/group.

In female GLP-1R^+/+^ mice, exendin-4 increased UCP-1 [F(1,18) = 5.967, *P*=0.025], Gpr120 [F(1,18) = 10.744, *P*=0.004], and Ppargc1a [F(1,18) = 19.767, *P*<0.001] while the effects of exendin-4 on thermogenic gene expression were absent in male and female GLP-1R^-/-^ mice.

**Figure 9:**
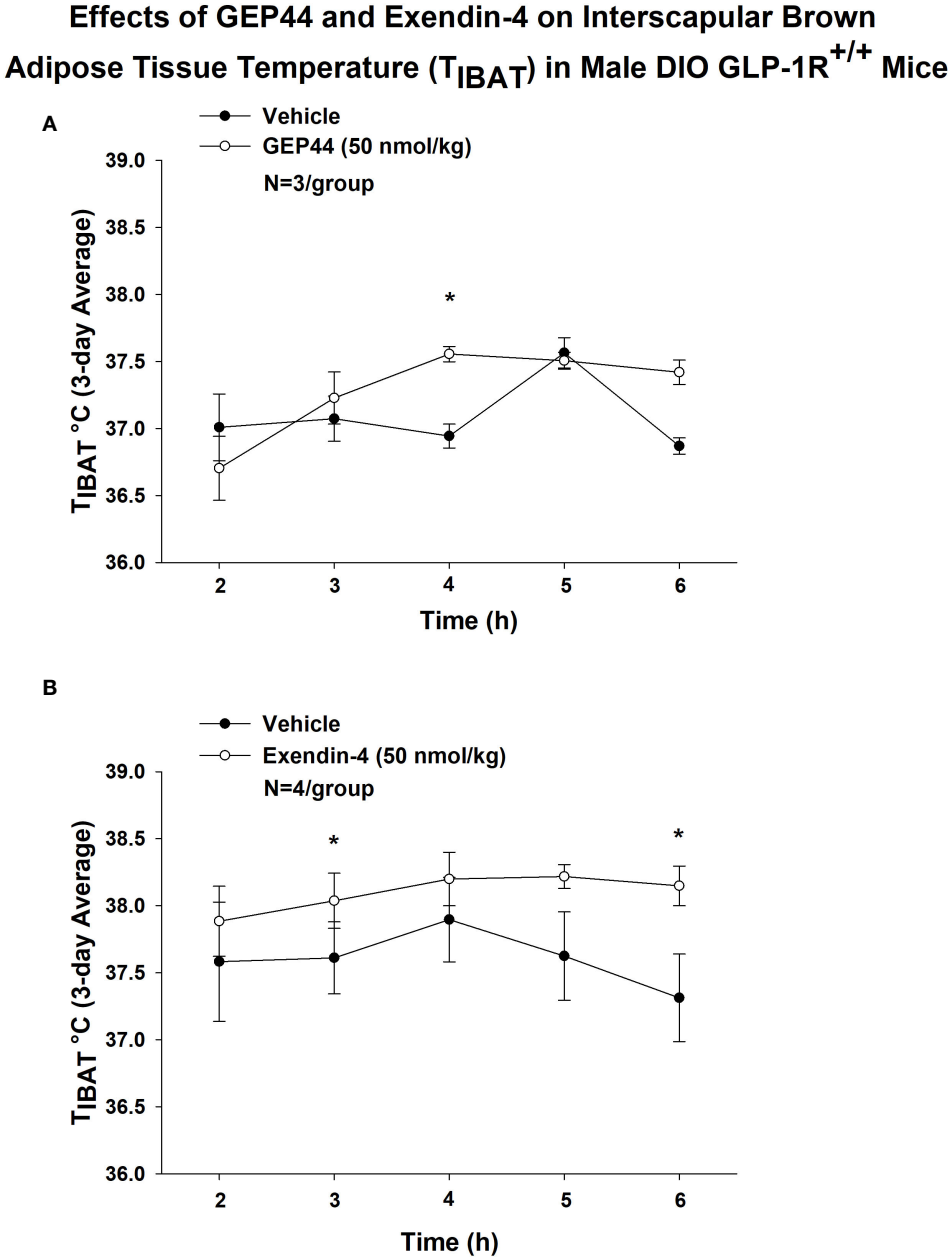
**(A, B)** Effects of GEP44 and exendin-4 on IBAT temperature (T_IBAT_) in male DIO GLP-1R^+/+^ mice. **(A)** Effect of GEP44 on T_IBAT_ in male DIO GLP-1R^+/+^ mice; **(B)** Effect of exendin-4 on T_IBAT_ in male DIO GLP-1R^+/+^ mice. T_IBAT_ data were collected at 2, 3, 4, 5 and 6-h post-injection and averaged throughout each sequential 3-day vehicle and 3-day drug treatment period. Data are expressed as mean ± SEM. *P<0.05 GEP44 or exendin-4 vs. vehicle.

We also implanted a subset of mice with temperature transponders (HTEC IPTT-300; BIO MEDIC DATA SYSTEMS, INC, Seaford, DE) underneath both IBAT pads in order to obtain a more functional measure of IBAT thermogenesis [IBAT temperature (T_IBAT_)] as previously described (17, 18). T_IBAT_ was measured at 2, 3, 4, 5 and 6-h post-injection and averaged throughout each sequential 3-day vehicle and 3-day drug treatment period. Note that T_IBAT_ was averaged across two-day vehicle treatments (prior to 20 and 50 nmol/kg) for one of the four cohorts used in the dose escalation studies. Similar to what we found with GEP44-elicited increases in IBAT thermogenic gene expression, we found that GEP44 (50 nmol/kg) also increased T_IBAT_ at 240-min post-injection in male DIO GLP-1R^+/+^ mice (N=3/group; *P*<0.05) (**Figure 9A**). Likewise, exendin-4 (50 nmol/kg) also increased T_IBAT_ at 180 and 360-min post-injection (N=4/group; *P*<0.05) (**Figure 9B**).

Lower doses of exendin-4 largely reproduced the effects found at the higher dose (50 nmol/kg). Namely, exendin-4 (10 nmol/kg) tended to increase T_IBAT_ at 300-min post-injection (*P*<0.05; data not shown) while the slightly higher dose (20 nmol/kg) increased T_IBAT_ at 240-min post-injection (*P*<0.05; data not shown). Exendin-4 (20 nmol/kg) appeared to produce a reduction of T_IBAT_ at 120-min post-injection (*P*<0.05; data not shown), but this was not observed at other doses. In contrast, GEP44 failed to produce significant effects on T_IBAT_ at lower doses (data not shown).

Together, our findings suggest that while both GEP44 and exendin-4 increase BAT thermogenesis, the effects of exendin-4 on BAT thermogenesis (T_IBAT_) appeared to be longer lasting relative to GEP44.

### Blood glucose and plasma hormones

**Figure 10:**
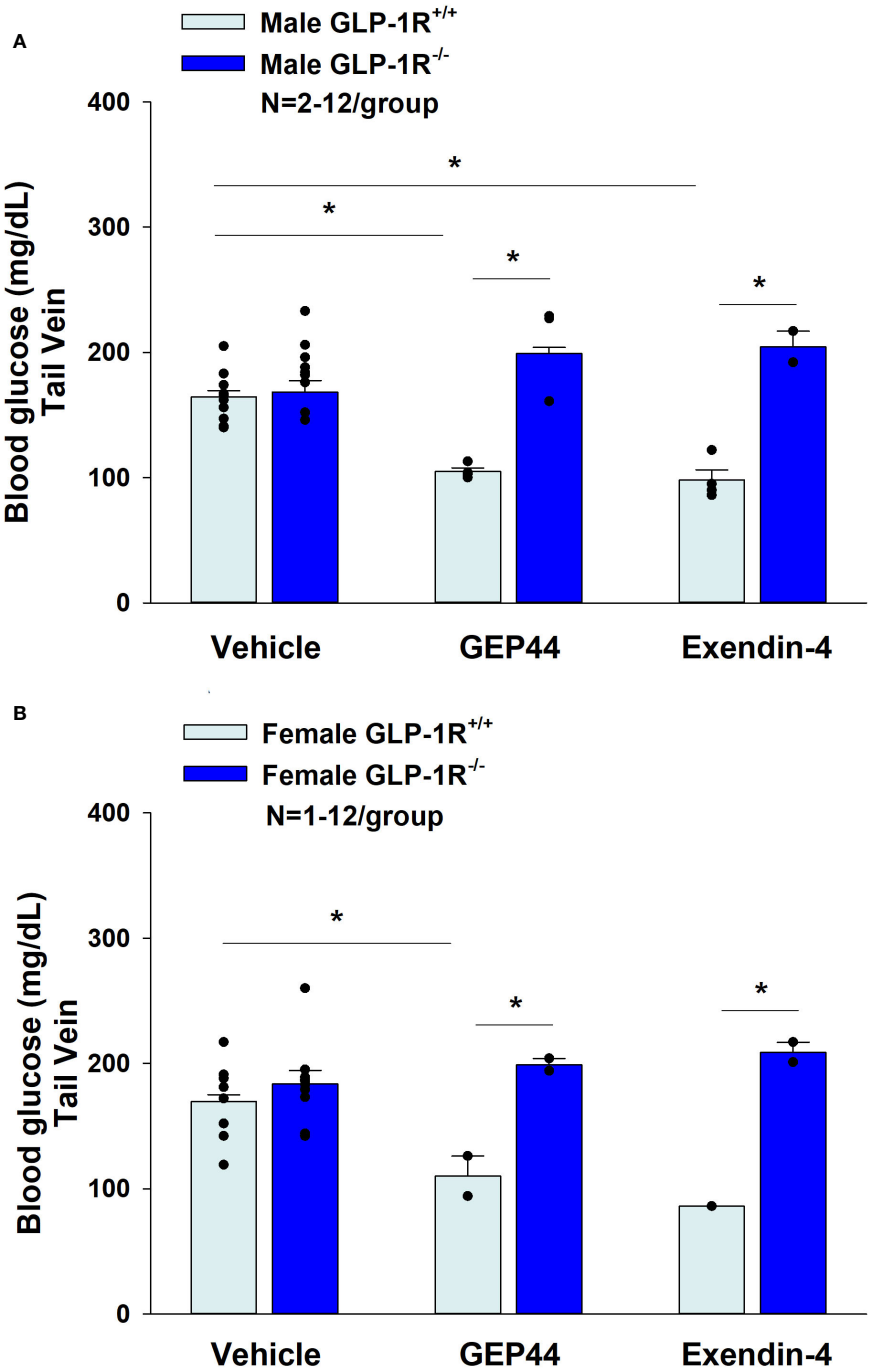
**(A, B)** Effects of GEP44 and exendin-4 on tail vein glucose in male and female DIO GLP-1R^+/+^ and GLP-1R^-/-^ mice. **(A)** Effect of GEP44 and exendin-4 on tail vein glucose in male DIO GLP-1R^+/+^ and GLP-1R^-/-^ mice; **(B)** Effect of GEP44 and exendin-4 on tail vein glucose in female DIO GLP-1R^+/+^ and GLP-1R^-/-^ mice. Blood was collected by tail vein nick (glucose) at 2-h post-injection of VEH, exendin-4 (50 nmol/kg) or GEP44 (50 nmol/kg). Tail vein glucose was measured at 2-h post-injection in conscious mice. Data are expressed as mean ± SEM. *P<0.05 GEP44 or exendin-4 vs. vehicle.

Consistent with previous findings in rats, we found that that GEP44 also reduced tail vein glucose (collected at 2-h post-injection) in both male (**Figure 10A**) [F(1,14) = 39.938, *P*<0.05)] and female GLP-1R^+/+^ DIO mice (**Figure 10B**) [F(1,10) = 6.954, *P*<0.05]. Moreover, the effects of GEP44 to reduce tail vein glucose were absent in both male [F(1,5) = 28.122, *P*<0.05] and female GLP-1R^-/-^ mice [F(1,2) = 28.189, *P*<0.05]. In addition, exendin-4 reduced fasting tail vein glucose in both male (**Figure 10B**) [F(1,14) = 41.690, *P*<0.05] and female GLP-1R^+/+^ DIO mice (**Figure 10B**) [F(1,9) = 7.241, *P*<0.05]. The reduction on blood glucose in response to GEP44 in both male and female GLP-1R^+/+^ mice appear to be mediated, at least in part, by a reduction of glucagon (**Tables 3A, B**). As was the case with GEP44, the effects of exendin-4 to reduce tail vein blood glucose were also blocked in male GLP-1R^-/-^ mice [F(1,4) = 54.475, *P*<0.05]. The effect of exendin-4 to reduce glucose also appeared to be impaired in female GLP-1R^-/-^ mice [F(1,1) = 78.797, *P*=0.071]. In addition, exendin-4 reduced plasma leptin in male DIO mice [F(1,12) = 8.522, *P*<0.05] (**Table 3A**) and tended to reduce leptin in female DIO mice [F(1,15) = 4.157, *P*=0.059] (**Table 3B**). Furthermore, GEP44 tended to reduce plasma leptin in female DIO mice [F(1,14) = 3.325, *P*=0.09] (**Table 3B**). In contrast, both GEP44 and exendin-4 failed to reduce plasma leptin in both male and female GLP-1R^-/-^ mice (*P*=NS). Both GEP44 [F(1,14) = 5.405, *P*<0.05] and exendin-4 [F(1,15) = 5.151, *P*<0.05] also reduced total cholesterol in female DIO mice (**Table 3B**). These effects on total cholesterol also tended to be observed in response to both exendin-4 [F(1,14) = 5.169, *P*<0.05] and GEP44 [F(1,13) = 3.805, *P*=0.073] in female GLP-1R^-/-^ mice (**Table 3B**).

Table 3(A, B) Effects of the chimeric peptide, GEP44 (50 nmol/kg) and the selective GLP-1R agonist, exendin-4 (50 nmol/kg), on plasma leptin, insulin, and total cholesterol in (A) male and (B) female DIO GLP-1R^+/+^ and GLP-1R^-/-^ mice.

**Table 3A:** Plasma Measurements Following SC Administration of GEP44 or Exendin-4 in Male DIO Mice.

	GLP-1R^+/+^			GLP-1R^-/-^		
SC Treatment	Vehicle	GEP44	Exendin-4	Vehicle	GEP44	Exendin-4
**Leptin (ng/mL)**	38.3 ± 3.7	29.8 ± 6.9	29.8 ± 6.0	36.1 ± 5.8	33.4 ± 4.8	34.5 ± 5.6
**Insulin (ng/mL)**	1.5 ± 0.5	4.4 ± 1.3*	1.5 ± 0.4	1.7 ± 0.5	1.5± 0.3	1.6 ± 0.5
**Glucagon (pmol/L)**	20.5 ± 5.9	6.1 ± 1.4*				
**Total Cholesterol (mg/dL)**	189.4 ± 23.2	159.1 ± 10.9	166.6 ± 11.4	186.0 ± 21.1	174.6 ± 6.7	184.7 ± 8.7

Blood was collected by tail vein nick (glucose) or cardiac stick (leptin, insulin, total cholesterol) at 2-h post-injection of VEH, exendin-4 (50 nmol/kg) or GEP44 (50 nmol/kg). Data are expressed as mean ± SEM. *P <0.05 GEP44 or exendin-4 vs. vehicle.N=6-10/group.

**Table 3B:** Plasma Measurements Following SC Administration of GEP44 or Exendin-4 in Female DIO Mice.

	GLP-1R^+/+^			GLP-1R^-/-^		
SC Treatment	Vehicle	GEP44	Exendin-4	Vehicle	GEP44	Exendin-4
**Leptin (ng/mL)**	34.1 ± 4.3	18.5 ± 5.1*	17.7 ± 4.7*	19.8 ± 2.7	15.2 ± 3.2	14.0 ± 4.1
**Insulin (ng/mL)**	1.4 ± 0.4	1.1 ± 0.3	1.0 ± 0.3	0.5 ± 0.1	0.5 ± 0.1	0.5 ± 0.1
**Glucagon (pmol/L)**	14.7 ± 3.0	5.5 ± 1.8*				
**Total Cholesterol (mg/dL)**	170.9 ± 18.0	116.5 ± 6.5*	122.9 ± 7.0*	132.7 ± 11.3	105.0 ± 6.4*	102.7 ± 7.7*

Blood was collected by tail vein nick (glucose) or cardiac stick (leptin, insulin, total cholesterol) at 2-h post-injection of VEH, exendin-4 (50 nmol/kg) or GEP44 (50 nmol/kg). Data are expressed as mean ± SEM. *P <0.05 GEP44 or exendin-4 vs. vehicle.N=7-10/group.

## Discussion

Here we report effects of the novel chimeric peptide (GEP44), which targets GLP-1R, Y1R and Y2R, on energy intake, BW, thermoregulation (core temperature) and gross motor activity in DIO mice and characterize the extent to which these effects are mediated through GLP-1R. We tested the hypothesis that GEP44 reduces energy intake and BW through a GLP-1R dependent mechanism. We found that GEP44 reduced BW in both male and female DIO male GLP-1R^+/+^ mice whereas these effects were absent in male and female DIO GLP-1R^-/-^ mice. These findings suggest that GLP-1R signaling contributes to GEP44-elicited reduction of BW in both male and female mice. Additionally, GEP44 decreased energy intake in both male and female DIO GLP-1R^+/+^ mice, but GEP44 produced more robust effects across multiple doses in males. These findings suggest that 1) GEP44 reduces BW, in part, through reductions in energy intake in DIO mice and 2) male mice might have enhanced sensitivity to the anorexigenic effects of GEP44. In GLP-1R^-/-^ mice, the effects on energy intake were observed only at low and high doses in males suggesting that GEP44 may reduce energy intake in males, in part, through a GLP-1R independent mechanism. GEP44 reduced both core temperature and activity in both male and female GLP-1R^+/+^ mice suggesting that reductions in energy expenditure and/or spontaneous activity-induced thermogenesis may also contribute to the weight lowering effects of GEP44 in mice. Lastly, we show that GEP44 reduced fasting blood glucose in DIO male and female mice through GLP-1R signaling. Together, these findings support the hypothesis that the chimeric peptide, GEP44, reduces energy intake, BW, core temperature, and glucose levels, in part, through a GLP-1R dependent mechanism.

We extend previous findings from our laboratory (13, 14) and demonstrate that the effects of GEP44 to elicit weight loss in both male and female mice are primarily driven by GLP-1R. These effects are mediated, at least in part, by reductions of energy intake. Similar to GLP-1R driven effects of GEP44 on BW, based on our findings in both male and female GLP-1R^-/-^ mice, the ability of GEP44 to reduce energy intake also appears to be largely mediated by GLP-1R. However, the finding that the low and high dose of GEP44 also reduced energy intake in male GLP-1R^-/-^ mice suggests other mechanisms are involved in contributing to these effects. Given the role of Y2R in the control of energy intake and BW (21), and results showing synergism/additive effects between co-administered GLP-1R and Y2-R agonists (22, 23), it is likely that the Y2R is also playing a role in the beneficial effects of GEP44 in terms of BW.

We incorporated the use of exendin-4 as a positive control for GLP-1R mediated effects on both food intake and BW. Similar to what others have reported (15, 24), we also report that exendin-4 reduced BW and energy intake in both male and female DIO mice through GLP-1R signaling. Exendin-4 treatment appeared to be more effective at reducing BW in males at the lowest dose (5 nmol/kg). These findings are consistent with previous findings from Baggio and colleagues who reported that a single dose of exendin-4 (1.5 μg or 0.356 nmol/mouse) failed to reduce chow diet intake in male GLP-1R^-/-^ mice (15). Another study also found that chronic subcutaneous administration of a single dose (0.126 mg/kg/day or 30 nmol/kg/day) also failed to reduce high fat diet intake and BW (vehicle corrected) in male GLP-1R^-/-^ mice (24). In contrast to our studies, only a single dose of exendin-4 was examined in male mice in both studies (15, 24) and neither study examined the effects of exendin-4 in male and female DIO mice.

We also extend previous findings from our laboratory (13, 14) to demonstrate that GEP44 reduces core temperature (surrogate measure of energy expenditure) in both male and female mice, similar to what we described earlier herein following exendin-4 treatment. These data are also consistent with a previous report by Hayes and colleagues showing that the GLP-1R agonist, exendin-4, produced a long-lasting reduction of core temperature (hypothermia) that lasted for 4 hours following systemic [intraperitoneal (IP)] injections in rats (25). The hypothermic effects may be due, in part, to the reduced thermic effect of food (diet-induced thermogenesis) in exendin-4 and GEP44 treated mice. Furthermore, Baggio and colleagues (15) found that central and peripheral (IP; 1.5 μg or 0.356 nmol/mouse) administration of exendin-4 reduced VO2 and resting energy expenditure in adult male wild-type mice. These effects occurred over 2 and 21 hours following peripheral and central administration, respectively. Similarly, Krieger and colleagues found that peripheral exendin-4 reduced energy expenditure over 4-h post-injection in rats (26). In addition, van Eyk and colleagues found that liraglutide treatment reduced resting energy expenditure after 4 weeks of treatment in humans with type 2 diabetes (27). Gabery initially found that semaglutide reduced energy expenditure during the dark phase in DIO mice through treatment day 6 but these differences were no longer significant after adjusting for lean mass (28). Similarly, Blundell and colleagues initially found that semaglutide reduced resting energy expenditure after 12 weeks of treatment in humans but these effects were no longer significant after adjusting for lean mass (29). Despite previous reports indicating that acute central (ICV) administration of GLP-1 increases core temperature over 4-h post-injection (30), IBAT temperature over 6-h post-injection (31) and activates SNS fibers that innervate BAT (31), to our knowledge, the majority of studies that administered GLP-1R agonists peripherally have either found a reduction or no change in energy expenditure. Given the dearth of studies that have measured both core temperature and energy expenditure in the same group of animals, it will be important to measure both endpoints simultaneously in the same animal model. Ongoing studies are currently in the process of addressing the extent to which systemic administration of GEP44 and exendin-4 alters core temperature and more directly impacts energy expenditure (measured by indirect calorimetry) in male and female DIO rats.

In contrast to the effects of GEP44 and exendin-4 to reduce core temperature, we found that both GEP44 and exendin-4 stimulated thermogenic markers (biochemical readout of BAT thermogenesis) within IBAT and T_IBAT_ (functional readout of BAT thermogenesis). It is not clear why GEP44 and exendin-4 elicited opposing effects on core temperature and T_IBAT_ given that BAT thermogenesis helps maintain core temperature in other contexts such as fever or stress (32, 33). Future studies that examine temporal profile will help identify if the elevated BAT thermogenesis may be in response to the reduction of core temperature. Our findings, however, that exendin-4 stimulated thermogenic markers within IBAT and IBAT temperature are consistent with what others have found following systemic (intraperitoneal) exendin-4 (34) or liraglutide (35) administration in mice. On the other hand, Krieger (26) found that systemic (IP) exendin-4 reduced BAT thermogenesis, skin temperature above the interscapular area over 2-h post-injection and BAT Adrb3 in a rat model. Note that measurements of skin temperature above the interscapular area might not necessarily reflect T_IBAT_ as the interscapular subcutaneous area may also be influenced by heating and cooling of the skin temperature. We found that the majority of thermogenic genes in IBAT were elevated in response to both GEP44 and exendin-4. Furthermore, our findings suggest that GEP44 may also elicit BAT thermogenesis through similar mechanisms (PPargc1a) in both male and female GLP-1R^+/+^ mice while exendin-4 may increase BAT thermogenesis via multiple thermogenic genes (UCP-1, Gpr120, and Ppargc1a) in female mice. In contrast to the effects of exendin-4 in GLP-1R^+/+^ mice, the chimeric peptide, GEP44, appears to promote BAT thermogenesis in both male and female GLP-1R^+/+^ mice through different genes. In contrast, GEP44 stimulated GPR120 only in female GLP-1R^-/-^ mice raising the possibility that other receptor subtypes may contribute to these effects at the high dose in female mice. Overall, our findings suggest that systemic administration of both GEP44 and exendin-4 may promote BAT thermogenesis through different mechanisms in a mouse model. It will be important in future studies to examine if SNS outflow to BAT is a predominant mediator of GEP44 or exendin-4-elicited weight loss in male and female DIO mice.

Our findings also demonstrate that GEP44 reduced gross motor activity through what appears to be both GLP-1R and Y2R signaling in male mice; while this effect appears to be entirely mediated by GLP-1R in female mice. In contrast, we found that exendin-4 treatment reduces activity strictly through GLP-1R in both male and female mice, as anticipated. Similarly, others have also found that exendin-4 at lower [0.5-5 μg/kg (0.2-2 μg/rat, IP)] (36) or higher doses [30 μg/kg (9 μg/rat, IP)] (37) reduces locomotor activity in lean male Sprague Dawley rats (36, 37). While Hayes and colleagues (25) did not find any change in activity in response to exendin-4 at [3 μg/kg (0.9 μg/rat, IP)] in lean male Sprague-Dawley rats, it might be possible that differences in paradigm and/or timing of injections may account for discrepancies between studies. Given that we found reductions in both core temperature and gross motor activity, our data raise the possibility that reductions in spontaneous physical activity-induced thermogenesis (38) and/or shivering and non-shivering thermogenesis in skeletal muscle (39) may have contributed to the hypothermic effects of both GEP44 and exendin-4. Moreover, our findings indicate that GEP44 may produce GLP-1R independent effects on 1) activity in male mice and 2) core temperature in both male and female mice while exendin-4 appears to impact both activity and core temperature through GLP-1R dependent mechanism in both male and female mice.

We have now extended our previous findings on glucoregulation (14) by showing that the effects of GEP44 to reduce fasting blood glucose in DIO male and female mice were mediated, in part, by GLP-1R. In addition, the effects of peripheral GEP44 and exendin-4 to reduce cholesterol in female GLP-1R^+/+^ and GLP-1R^-/-^ mice are also consistent with the potential GLP-1R and/or Y2-receptor mediated cholesterol lowering effects observed following the GLP-1R agonists, liraglutide (40) and exendin-4 (41, 42), Y2 receptor agonist, lipidated PYY_3-36_ analog, [Lys_7_(C16- γGlu)PYY3−36] (similar potency on Y2R as PYY_3-36_) (40), and dual GLP-1R/Y2 receptor agonists, 6q and peptide 19 (40), in DIO rodents.

One limitation to our studies is the lack of weight restricted or pair-fed controls for studies that assessed the effects of GEP44 and exendin-4 on gene expression as well as plasma measurements (total cholesterol, leptin, and glucose) in adult DIO male and female mice. As a result, it is possible that the effects of GEP44 and exendin-4 on thermogenic gene expression or glucose, total cholesterol or leptin lowering in DIO mice may also be due, in part, to the weight loss in response to GEP44 or exendin-4 treatment. In addition, we only collected T_IBAT_ between 120 and 360 min-post-injection. It is possible that, due to absence of being able to measure T_IBAT_ at earlier time points, we might have missed any potential hypothermic effects that might have preceded the elevations of T_IBAT_ that occurred at the later time points. In addition, we were unable to assess the roles of Y1R or Y2R signaling more fully in contributing to the effects of GEP44. Future studies incorporating the use of Y2R deficient mice will be helpful in more fully establishing the role of Y2R signaling in the anorectic response to GEP44.

In summary, the results presented in this manuscript highlight the beneficial effects of the dual-agonist, GEP44, on BW, energy intake, and glucoregulation in adult male and female DIO mice at doses that have not been found to elicit visceral illness in rats or emesis in shrews (13, 14). Our findings further demonstrate that effects on changes of BW, energy intake, activity, and glucose levels are largely mediated through GLP-1R signaling. However, given that GEP44 may also elicit GLP-1R independent effects on 1) activity in male mice and 2) core temperature in both male and female mice, it is possible that other receptor subtypes, including the Y1R (43) and Y2R, may contribute to these effects. The findings to date indicate that GEP44 is a promising drug targeting limitations associated with current GLP-1R agonist medications (5, 44) for the treatment of obesity and/or T2DM.

## Data availability statement

All relevant data is contained within the article: The original contributions presented in the study are included in the article/**Supplementary Material**, further inquiries can be directed to the corresponding author/s.

## Ethics statement

The animal study was approved by VA Puget Sound Health Care System IACUC and the US Army Medical Research and Development Command (USAMRDC) Animal Care and Use Review Office (ACURO). The study was conducted in accordance with the local legislation and institutional requirements.

## Author contributions

JB: Writing – review & editing, Writing – original draft, Supervision, Resources, Project administration, Methodology, Investigation, Formal analysis, Data curation, Conceptualization. MH: Writing – review & editing, Writing – original draft, Resources, Project administration, Methodology, Investigation, Data curation. JS: Writing – review & editing, Writing – original draft, Resources, Project administration, Methodology, Investigation, Data curation. MG: Writing – review & editing, Writing – original draft, Resources, Project administration, Methodology, Investigation, Data curation. JR: Writing – review & editing, Writing – original draft, Resources, Project administration, Methodology, Investigation, Data curation. ET: Writing – review & editing, Writing – original draft, Resources, Project administration, Methodology, Investigation, Data curation. AD: Writing – review & editing, Writing – original draft, Resources, Project administration, Methodology, Investigation, Data curation. KS: Writing – review & editing, Writing – original draft, Project administration, Investigation. TS: Writing – review & editing, Writing – original draft, Methodology, Investigation, Conceptualization. CE: Writing – review & editing, Writing – original draft, Methodology, Investigation, Funding acquisition, Conceptualization. KC: Writing – review & editing, Writing – original draft, Resources, Methodology, Investigation. EA: Writing – review & editing, Writing – original draft, Resources, Methodology, Investigation. SZ: Writing – review & editing, Writing – original draft, Resources, Project administration, Methodology, Investigation. RD: Writing – review & editing, Writing – original draft, Supervision, Resources, Project administration, Methodology, Investigation, Funding acquisition, Conceptualization. CR: Writing – review & editing, Writing – original draft, Supervision, Resources, Project administration, Methodology, Investigation, Funding acquisition, Conceptualization.

## Supplementary material

Supplementary Figure 1
**(A–D)** Effects of the chimeric peptide, GEP44, on energy intake (kcal/gram BW) in male and female DIO GLP-1R^+/+^ and GLP-1R^-/-^ mice. Mice were maintained on HFD (60% kcal from fat; N=10-11/group) for approximately 4 months prior to receiving SC injections of vehicle (sterile saline/water) followed by escalating doses of GEP44 (5, 10, 20 and 50 nmol/kg; 3 mL/kg injection volume). **(A)** Effect of GEP44 on energy intake in male HFD-fed DIO GLP-1R^+/+^ mice; **(B)** Effect of GEP44 on change on energy intake in male HFD-fed DIO GLP-1R^-/-^ mice; **(C)** Effect of GEP44 on energy intake in male HFD-fed DIO GLP-1R^+/+^ mice; **(D)** Effect of GEP44 on energy intake in male HFD-fed DIO GLP-1R ^-/-^ mice. Energy intake (kcal/gram BW) was averaged throughout each sequential 3-day vehicle and 3-day drug treatment period. Data are expressed as mean ± SEM. **P*<0.05 GEP44 vs. vehicle.

Supplementary Figure 2
**(A–D)** Effects of the selective GLP-1R agonist, exendin-4, on energy intake (kcal/gram BW) in male and female DIO GLP-1R^+/+^ and GLP-1R^-/-^ mice. Mice were maintained on HFD (60% kcal from fat; N=9-11/group) for approximately 4 months prior to receiving SC injections of vehicle followed by escalating doses of GEP44 (5, 10, 20 and 50 nmol/kg; 3 mL/kg injection volume). **(A)** Effect of GEP44 on energy intake in male HFD-fed DIO GLP-1R^+/+^ mice; **(B)** Effect of GEP44 on change on energy intake in male HFD-fed DIO GLP-1R^-/-^ mice; **(C)** Effect of GEP44 on energy intake in male HFD-fed DIO GLP-1R^+/+^ mice; **(D)** Effect of GEP44 on energy intake in male HFD-fed DIO GLP-1R^-/-^ mice. Energy intake (kcal/gram BW) was averaged throughout each sequential 3-day vehicle and 3-day drug treatment period. Data are expressed as mean ± SEM. **P*<0.05 exendin-4 vs. vehicle.
