# Supplementary figures and images for: The novel chimeric multi-agonist peptide (GEP44) reduces energy intake and body weight in male and female diet-induced obese mice in a glucagon-like peptide-1 receptor-dependent manner
Source: Front Endocrinol (Lausanne). 2024 Jul 22;15:1432928. doi: 10.3389/fendo.2024.1432928 (PMC11298355; doi:10.3389/fendo.2024.1432928)

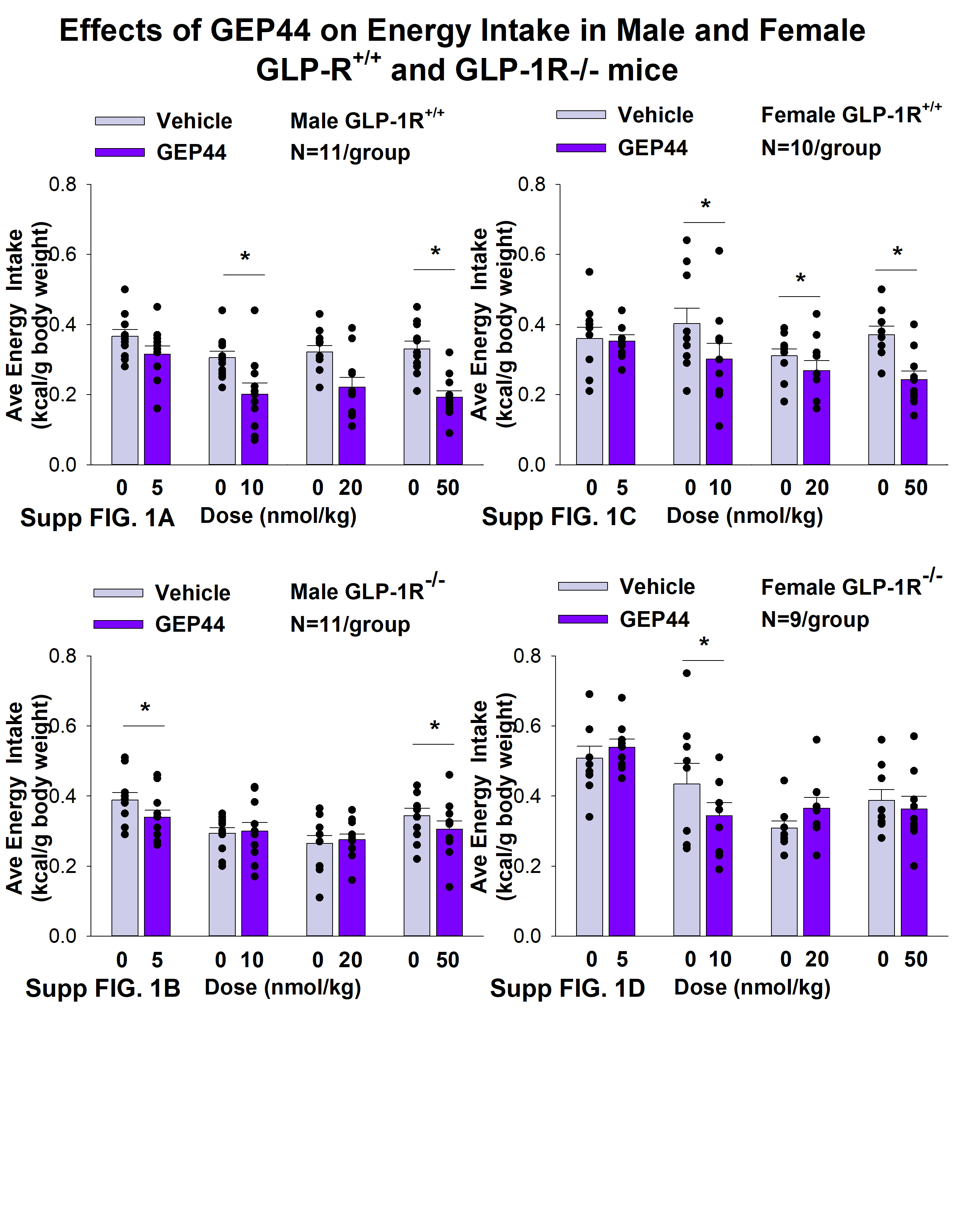

Supplement: Supplementary Figure 1 — (A–D) Effects of the chimeric peptide, GEP44, on energy intake (kcal/gram BW) in male and female DIO GLP-1R+/+ and GLP-1R-/- mice. Mice were maintained on HFD (60% kcal from fat; N=10-11/group) for approximately 4 months prior to receiving SC injections of vehicle (sterile saline/water) followed by escalating doses of GEP44 (5, 10, 20 and 50 nmol/kg; 3 mL/kg injection volume). (A) Effect of GEP44 on energy intake in male HFD-fed DIO GLP-1R+/+ mice; (B) Effect of GEP44 on change on energy intake in male HFD-fed DIO GLP-1R-/- mice; (C) Effect of GEP44 on energy intake in male HFD-fed DIO GLP-1R+/+ mice; (D) Effect of GEP44 on energy intake in male HFD-fed DIO GLP-1R -/- mice. Energy intake (kcal/gram BW) was averaged throughout each sequential 3-day vehicle and 3-day drug treatment period. Data are expressed as mean ± SEM. *P<0.05 GEP44 vs. vehicle. [file Image_1.jpeg]

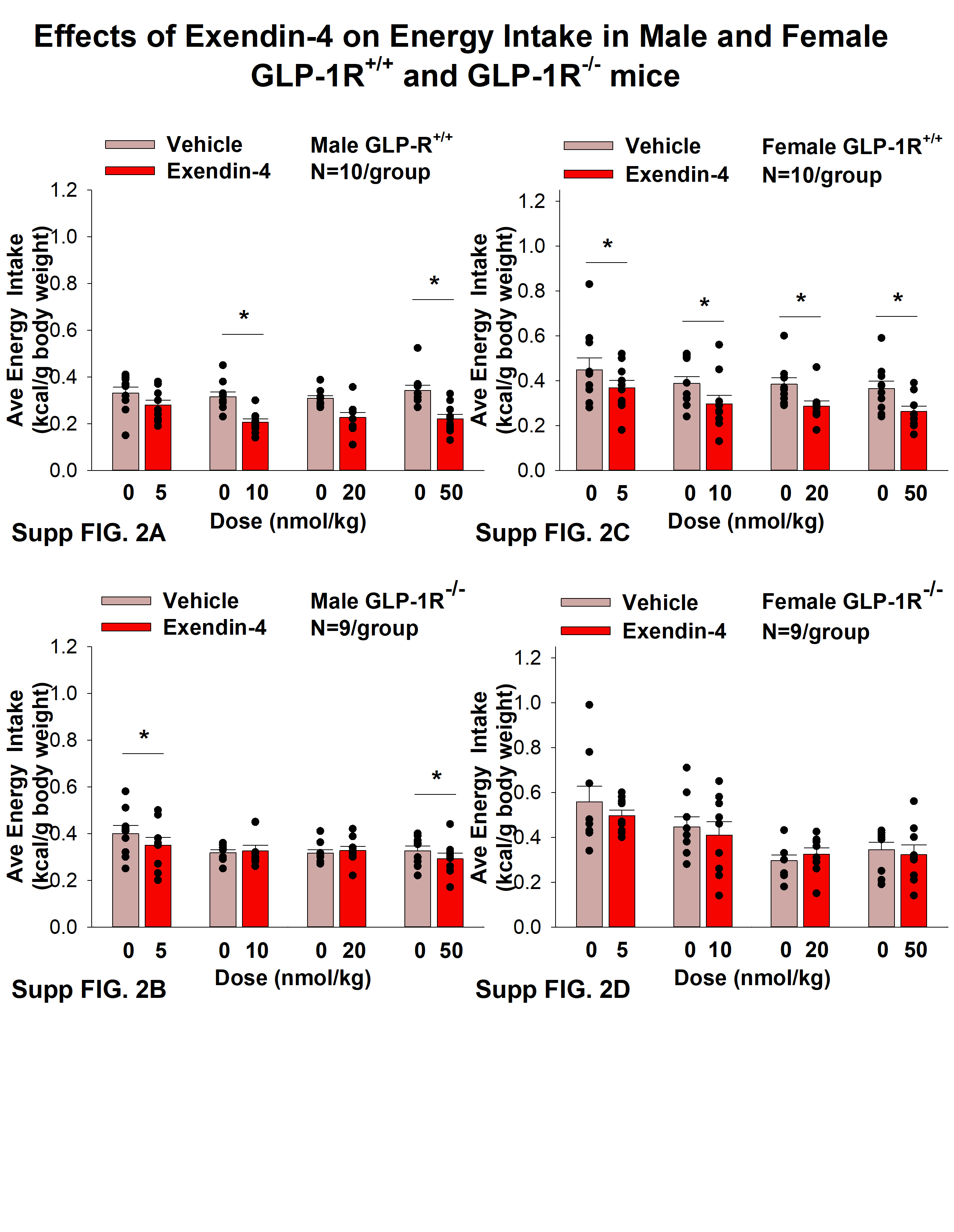

Supplement: Supplementary Figure 2 — (A–D) Effects of the selective GLP-1R agonist, exendin-4, on energy intake (kcal/gram BW) in male and female DIO GLP-1R+/+ and GLP-1R-/- mice. Mice were maintained on HFD (60% kcal from fat; N=9-11/group) for approximately 4 months prior to receiving SC injections of vehicle followed by escalating doses of GEP44 (5, 10, 20 and 50 nmol/kg; 3 mL/kg injection volume). (A) Effect of GEP44 on energy intake in male HFD-fed DIO GLP-1R+/+ mice; (B) Effect of GEP44 on change on energy intake in male HFD-fed DIO GLP-1R-/- mice; (C) Effect of GEP44 on energy intake in male HFD-fed DIO GLP-1R+/+ mice; (D) Effect of GEP44 on energy intake in male HFD-fed DIO GLP-1R-/- mice. Energy intake (kcal/gram BW) was averaged throughout each sequential 3-day vehicle and 3-day drug treatment period. Data are expressed as mean ± SEM. *P<0.05 exendin-4 vs. vehicle. [file Image_2.jpeg]
